# Diagnostic and therapeutic effects of fluorescence cystoscopy and narrow-band imaging in bladder cancer: a systematic review and network meta-analysis

**DOI:** 10.1097/JS9.0000000000000592

**Published:** 2023-08-01

**Authors:** Zhinan Fan, Hongjin Shi, Jiayu Luo, Xinquan Guo, Bo Wang, Yao Liu, Junjie Yu

**Affiliations:** a Department of Urology , Meishan People’s Hospital, Meishan; b Department of Urology, The Second Affiliated Hospital of Kunming Medical University, Kunming, People’s Republic of China

**Keywords:** bladder cancer, detection rate, fluorescence cystoscopy, narrow-band imaging, network meta-analysis, recurrence rate

## Abstract

**Background:**

This review aims to compare the efficacies of fluorescence cystoscopy, narrow-band imaging (NBI), and white light cystoscopy in the treatment and diagnosis of bladder cancer.

**Methods:**

The authors searched PubMed, EMbase, Web of Science, and the Cochrane Library from January 1990 to April 2022. A total of 26 randomized controlled studies and 22 prospective single-arm studies were selected. Most patients had nonmuscle-invasive bladder cancer. The study protocol has been registered at PROSPERO.

**Results:**

In the pairwise meta-analysis, 5-aminolevulinic acid (5-ALA) reduced the short-term and long-term recurrence rates of bladder cancer compared with white light cystoscopy (WLC); however, no statistical difference was observed in intermediate-term recurrence rates (RR=0.79, 95% CI: 0.57–1.09). Hexaminolevulinic acid and NBI reduced short-term, intermediate-term, and long-term recurrence rates. The sensitivity of 5-ALA, hexaminolevulinic acid, NBI, and WLC for bladder cancer were 0.89 (95% CI: 0.81–0.94), 0.96 (95% CI: 0.92–0.98), 0.96 (95% CI: 0.92–0.98), and 0.75 (95% CI: 0.70–0.79), respectively; however, only NBI had the same specificity as WLC (0.74 vs. 0.74). Compared with WLC, 5-ALA improved the detection rate of carcinoma in situ and Ta stage bladder cancer but had no advantage in T1 stage tumors (OR=2.39, 95% CI:0.79–7.19). Hexaminolevulinic acid and NBI improved the detection rates of all nonmuscular-invasive bladder cancers. In the network meta-analysis, there was no significant difference in either recurrence or detection rates between 5-ALA, hexaminolevulinic acid, and NBI.

**Conclusion:**

Fluorescence cystoscopy and NBI are advantageous for treating and diagnosing patients with nonmuscle-invasive bladder cancer.

## Introduction

Highlights

- The advantages of fluorescence cystoscopy (FC) and narrow-band imaging (NBI) are higher in patients with nonmuscle-invasive bladder cancer.
- Compared with white light cystoscopy (WLC), FC and NBI reduced the recurrence rate of bladder cancer.
- Compared with WLC, FC and NBI showed higher sensitivity but lower specificity, only NBI had the same specificity as WLC.
- FC and NBI may significantly improve the detection rate of nonmuscle-invasive bladder cancer, especially carcinoma in situ.
- Compared with WLC, FC and NBI did not improve the detection rate of muscle-invasive bladder cancer.

Bladder cancer is a common urinary system tumor with an increasing incidence globally. Currently, the burden of bladder cancer is concentrated in developed countries, where the incidence is approximately three times higher than that in developing countries (9.5 vs. 3.3)^1^. Bladder cancers can be divided into muscle-invasive and nonmuscle-invasive carcinomas. Transurethral resection of bladder tumor (TURBT) is the main treatment for nonmuscle-invasive bladder cancer; however, the postoperative recurrence rate is relatively high^2^. TURBT is mainly performed intraoperatively with white light cystoscopy (WLC) to identify the tumor with the naked eye, which inevitably leads to some omissions, especially in carcinoma in situ (CIS). Such missed detection can lead to a significant recurrence. Fluorescence cystoscopy (FC) and narrow-band imaging (NBI) techniques have been developed to improve detection rates. FC aggregates in bladder cancer tissues with 5-aminolevulinic acid (5-ALA) or hexaminolaevu-linate (HAL) producing fluorescent substances, thus forming a clear contrast between tumors and normal bladder mucosa^3^. In NBI, the white light of the traditional cystoscope is replaced by narrow green and blue light so that the capillaries of the mucosal surface can be displayed clearly, which also differentiates normal tissue from tumor^4^. We performed a pairwise meta-analysis and network meta-analysis (NMA) to evaluate the diagnostic and therapeutic performance of 5-ALA, HAL, NBI, and WLC in bladder cancer and to rank different treatment regimens for reference.

## Methods

This study was constructed from the PRISMA (Preferred Reporting Items for Systematic Reviews and Meta-Analyses) (Supplemental Digital Content 1, http://links.lww.com/JS9/A797) (Supplemental Digital Content 2, http://links.lww.com/JS9/A798) and AMSTAR (Assessing the methodological quality of systematic reviews) Guidelines^5^ (Supplemental Digital Content 3, http://links.lww.com/JS9/A799). And the study protocol has been registered at PROSPERO.

### Criteria for inclusion

Participants: Patients diagnosed with urothelial carcinoma of the bladder.

Intervention and comparison: The experimental group used FC or NBI, whereas the control group used WLC.

Results: Primary results: recurrence rate (short-term: ≤3 months, intermediate-term: 3 months–1 year, long-term: >1 year), sensitivity, specificity, and detection rate (Ta, T1, CIS, muscle-invasive bladder cancer). Secondary results: progression rate (short-term: ≤1 year, long-term: >1 year), positive likelihood ratio (PLR), negative likelihood ratio (NLR), and diagnostic odds ratio (DOR).

Study design: Randomized controlled studies were included in the treatment analysis, and prospective single-arm studies were included in the diagnostic analysis.

### Exclusion criteria

The exclusion criteria were as follows: patients with other types of bladder neoplasms (adenocarcinoma, squamous cell carcinoma, upper urinary tract tumor, or secondary bladder cancer) and repeated detection and published literature.

### Search strategy

Literature searches were conducted in PubMed, EMbase, the Web of Science, and the Cochrane Library from January 1990 to April 2022. Keywords were bladder cancer, FC, and NBI.

### Data extraction

Two researchers independently conducted literature screening and data extraction according to the inclusion and exclusion criteria, and differences were resolved through discussion or with the assistance of a third researcher. The following information was extracted from the literature that met the inclusion criteria: author and publication time of the included literature; sample size and specific operating conditions of the experimental and control groups; extraction of outcome indicators. Finally, both sides were cross-checked.

### Quality assessment

Two investigators conducted independent quality assessments based on the Cochrane Bias Risk Tool (for randomized controlled studies) and QUADAS-2 (for prospective studies).

### Statistical analysis

A pairwise meta-analysis was performed using Stata15.1 software. A heterogeneity test was conducted for the included studies. If *P*>0.1 and *I* ^2^<50%, indicating no heterogeneity, a fixed-effect model was adopted; otherwise, the random-effects model was adopted, and the possible sources of heterogeneity were analyzed. Related forest maps were drawn to obtain the final results. In addition, a summary receiver operating characteristic (SROC) curve was constructed for diagnostic analysis. The area under the SROC curve (AUC) was calculated. We used R 4.1 software to conduct a Bayesian NMA. All efficacy assessments were analyzed using random or fixed-effects models, and the most appropriate model was selected according to the lowest Bayesian deviance information criterion (DIC). For example, if the DIC of the random-effects model is lower than that of the fixed-effects model by more than five, the random-effects model was chosen. The effects of the intervention were ranked according to the surface under the cumulative ranking curve (SUCRA): the higher the SUCRA value, the higher the ranking, indicating that the intervention was likely to be most successful. Statistical significance was set at *P*<0.05. Funnel plots were drawn for meta-analysis and NMA analyses containing at least 10 original studies^6^, and Egger’s test were used to check the asymmetry of the funnel plots.

## Results

### Search results and study characteristics

A total of 1069 relevant literatures were obtained in the initial examination, and 48 studies were included after layer-by-layer screening. The literature screening process and results are illustrated in Supplementary Fig. 1 (Supplemental Digital Content 4, http://links.lww.com/JS9/A800). Most patients included in the study had nonmuscle-invasive bladder cancer. There were 26 randomized clinical trials (RCTs)^7-34^, of which six were multicenter trials, five were double-center trials, one was double-blind, and one was single-blind. Seven studies compared 5-ALA with WLC, 10 compared HAL with WLC, and 9 compared NBI with WLC. In addition, Riedl, Filbeck, and Stenzl published long-term follow-up results for many years after the initial study was published, which were included in this analysis^35-37^. The baseline characteristics of the included studies are summarized in Supplementary Table 1 (Supplemental Digital Content 4, http://links.lww.com/JS9/A800).

In addition, 22 prospective non-RCTs^38-57^ (all single-arm studies) were compared in the same patient group. Eleven studies were multicenter clinical trials, one was a double-center clinical trial, five studies compared 5-ALA with WLC, twelve compared HAL with WLC, and five compared NBI with WLC. The baseline characteristics of the included studies are summarized in Supplementary Table 2 (Supplemental Digital Content 4, http://links.lww.com/JS9/A800).

### Quality assessment

The assessment of bias for RCT and prospective single-arm studies are illustrated in Supplementary Figs 2–5 (Supplemental Digital Content 4, http://links.lww.com/JS9/A800).

### Recurrence rate

**Figure 1:**
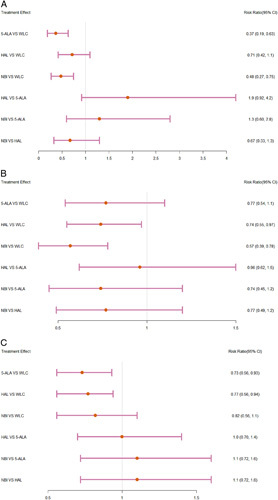
Forest plots of recurrence rate of bladder cancer in network meta-analysis. (A) Short-term recurrence rate. (B) Intermediate-term recurrence rate. (C) Long-term recurrence rate. 5-HAL, 5-aminolevulinic acid; HAL, hexaminolaevu-linate; NBI, narrow-band imaging; WIC, white light cystoscopy.

Short-term recurrence rate: In the pairwise meta-analysis, 5-ALA, HAL, and NBI reduced the short-term recurrence rate compared with WLC (5-ALA: RR=0.36, 95% CI: 0.20–0.68; HAL: RR=0.79, 95% CI: 0.65–0.95; NBI: RR=0.47, 95% CI: 0.29–0.77). As illustrated in Supplementary Fig. 6 (Supplemental Digital Content 4, http://links.lww.com/JS9/A800). The NMA analysis showed no statistically significant differences between 5-ALA, HAL, and NBI (Fig. 1A). According to the SUCRA results, the effectiveness rates from highest to lowest were 5-ALA (90.99%), NBI (70.70%), HAL (36.17%), and WLC (2.15%).

Intermediate-term recurrence rate: In the pairwise meta-analysis, HAL and NBI reduced the intermediate-term recurrence rate compared with WLC (HAL: RR=0.79, 95% CI: 0.71–0.88; NBI: RR=0.55, 95% CI: 0.37–0.81); however, 5-ALA did not reveal a statistical difference (Supplementary Fig. 7, Supplemental Digital Content 4, http://links.lww.com/JS9/A800). The NMA analysis showed no statistically significant differences between 5-ALA, HAL, and NBI (Fig. 1B). According to the results of SUCRA, the effective rates from highest to lowest were NBI (93.55%), HAL (56.01%), 5-ALA (48.27%), and WLC (2.17%).

Long-term recurrence rate: In the pairwise meta-analysis, 5-ALA, HAL, and NBI reduced the long-term recurrence rate compared to WLC (5-ALA: RR=0.72, 95% CI: 0.62–0.84; HAL: RR=0.75, 95% CI: 0.60–0.93; NBI: RR=0.80, 95% CI: 0.66–0.97). As illustrated in Supplementary Fig. 8 (Supplemental Digital Content 4, http://links.lww.com/JS9/A800). The NMA analysis showed no statistically significant differences between 5-ALA, HAL, and NBI (Fig. 1C). According to the results of SUCRA, the effective rates from highest to lowest were 5-ALA (77.14%), HAL (67.88%), NBI (51.94%), and WLC (3.04%).

Subgroup analysis: Among the 26 literatures included in the analysis of recurrence rate, 3 literatures clearly stated that there was no intravesical chemotherapy after TURBT. After excluding these three literatures, a meta-analysis was conducted again and found that the relevant results remained unchanged (Supplementary Figs 9–11, Supplemental Digital Content 4, http://links.lww.com/JS9/A800). In addition, there are 13 literatures that have second TURBT for patients with insufficient TURBT for the first time or without muscle tissue in postoperative specimens. A separate meta-analysis of these 13 literatures showed that the results of the related meta-analysis of ALA have not changed. HAL has no statistical difference in reducing the short-term and intermediate-term recurrence rate of bladder cancer, but there is still a significant difference in the long-term recurrence rate; NBI still has statistical differences in reducing the short-term and intermediate-term recurrence rate of bladder cancer, but there is no statistical difference in the long-term recurrence rate (Supplementary Figs 12–14, Supplemental Digital Content 4, http://links.lww.com/JS9/A800).

### Sensitivity and specificity

**Figure 2:**
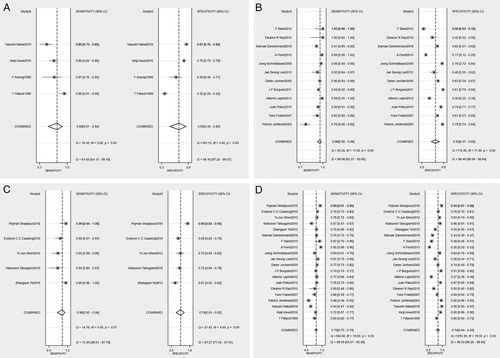
Forest plots of specificity and sensitivity of FC, NBI, and WLC for detection of bladder cancer. (A) 5-ALA; (B) HAL; (C) NBI; (D) WLC. 5-ALA, 5-aminolevulinic acid; HAL, hexaminolaevu-linate; NBI, narrow-band imaging; WIC, white light cystoscopy.

At the lesion level, the aggregate sensitivities of 5-ALA, HAL, NBI, and WLC were 0.89 (95% CI: 0.81–0.94), 0.96 (95% CI: 0.92–0.98), 0.96 (95% CI: 0.92–0.98), and 0.75 (95% CI: 0.70–0.79), respectively. The aggregate specificities were 0.65 (95% CI: 0.45–0.80), 0.53 (95% CI: 0.37–0.69), 0.74 (95% CI: 0.63–0.82), and 0.74 (95% CI: 0.64–0.83), respectively. As illustrated in Figure 2. The AUCs were 0.88 (95% CI: 0.85–0.90), 0.91 (95% CI: 0.88–0.93), 0.95 (95% CI: 0.92–0.96), and 0.80 (95% CI: 0.76–0.83), respectively. As illustrated in Supplementary Fig. 15 (Supplemental Digital Content 4, http://links.lww.com/JS9/A800). The PLRs were 2.5 (95% CI: 1.6–4.0), 2.1(95% CI: 1.5–2.9), 3.7(95% CI: 2.5–5.4), and 0.80 (95% CI: 0.76–0.83), respectively. The NLRs were 0.17 (95% CI: 0.12–0.24), 0.07 (95% CI: 0.04–0.13), 0.05 (95% CI: 0.02–0.11), and 0.80 (95% CI: 0.76–0.83), respectively. The DORs were 15 (95% CI: 10–22), 27 (95% CI: 16–48), 71 (95% CI: 27–189), and 0.80 (95% CI: 0.76–0.83), respectively.

### Detection rate

**Figure 3:**
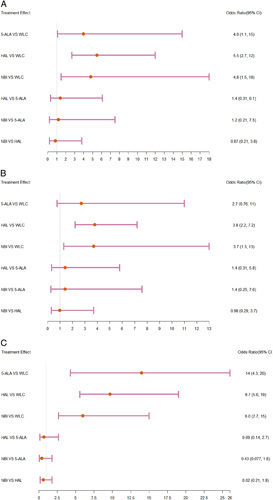
Forest plots of detection rate of bladder cancer in network meta-analysis. (A) Ta stage tumors; (B) T1 stage tumors; (C) CIS. 5-ALA, 5-aminolevulinic acid; CIS, carcinoma in situ; HAL, hexaminolaevu-linate; NBI, narrow-band imaging; WIC, white light cystoscopy.

Ta stage tumors: In the pairwise meta-analysis, 5-ALA, HAL, and NBI increased the detection rate of Ta stage bladder tumors compared with WLC (5-ALA: OR=3.81, 95% CI: 2.19–6.63; HAL: OR=4.78, 95% CI: 2.71–8.43; NBI: OR=4.18, 95% CI: 1.80–9.71). As illustrated in Supplementary Fig. 16 (Supplemental Digital Content 4, http://links.lww.com/JS9/A800). In NMA, there was no statistical difference between 5-ALA, HAL, and NBI (Fig. 3A). According to the SUCRA results, the detection rates from highest to lowest were HAL (75.43%), NBI (66.25%), 5-ALA (57.45%), and WLC (0.87%).

T1 stage tumors: In the pairwise meta-analysis, HAL and NBI increased the detection rate of T1 stage bladder tumors compared with WLC (HAL: OR=3.58, 95% CI: 2.21–5.80; NBI: OR=3.39, 95% CI: 1.33–8.66); however, 5-ALA did not reveal a statistical difference. As illustrated in Supplementary Fig. 17 (Supplemental Digital Content 4, http://links.lww.com/JS9/A800). In NMA, there was no statistical difference between 5-ALA, HAL, and NBI (Fig. 3B). According to the SUCRA results, the detection rates from highest to lowest were HAL (72.98%), NBI (71.05%).

CIS: In the pairwise meta-analysis, 5-ALA, HAL, and NBI increased the CIS detection rate compared to WLC (5-ALA: OR=8.77, 95% CI: 4.54–16.94; HAL: OR=8.15, 95% CI: 5.32–12.50; NBI: OR=5.38, 95% CI: 3.25–8.90). As illustrated in Supplementary Fig. 18 (Supplemental Digital Content 4, http://links.lww.com/JS9/A800). In NMA, there was no statistical difference between 5-ALA, HAL, and NBI (Fig. 3C). According to the SUCRA results, the detection rates from highest to lowest were 5-ALA (86.19%), HAL (70.68%), NBI (43.13%), and WLC (0.0075%).

Muscle-invasive bladder cancer: In the pairwise meta-analysis, 5-ALA, HAL, or NBI did not increase the detection rate of muscle-invasive bladder cancer compared to WLC (Supplementary Fig. 19, Supplemental Digital Content 4, http://links.lww.com/JS9/A800).

### Progression rate

Short-term progression rate: In the pairwise meta-analysis, 5-ALA, HAL, or NBI did not reduce the short-term progression rate compared to WLC (Supplementary Fig. 20, Supplemental Digital Content 4, http://links.lww.com/JS9/A800).

Long-term progression rate: In the pairwise meta-analysis, 5-ALA and HAL reduced the long-term progression rate compared with WLC (5-ALA: RR=0.48, 95% CI: 0.26–0.90; HAL: RR=0.56, 95% CI: 0.32–0.99); however, NBI did not reveal a statistical difference (Supplementary Fig. 21, Supplemental Digital Content 4, http://links.lww.com/JS9/A800).

### Publication bias tests

In the recurrence rate analysis, the number of original studies included in each pairwise meta-analysis was less than 10, so no funnel plot was drawn, and potential publication bias was discovered in the NMA (Supplementary Fig. 22, Supplemental Digital Content 4, http://links.lww.com/JS9/A800). However, in the detection rate analysis, neither meta-analysis nor NMA revealed publication bias (Supplementary Fig. 23-24, Supplemental Digital Content 4, http://links.lww.com/JS9/A800).

## Discussion

The high recurrence rate of bladder cancer after primary TURBT has always been a challenge in urinary tumor treatment. The initial TURBT is insufficient, resulting in a residual tumor at the margin. In addition, flat and small tumor lesions are easily ignored during surgery^20^. Therefore, FC and NBI have emerged as novel assistive technologies.

In normal cells, 5-ALA and HAL produce protoporphyrin IX (PpIX), subsequently catalyzed by ferrochelatase for ferrous iron insertion into PpIX to form heme and bilirubin. However, in tumor cells, the low activity of ferrochelatase, coupled with the activation of porphyrin synthetic enzyme and peptide transporter 1, promotes the production of PpIX, which leads to the accumulation of PpIX in cancer tissues^3,58^. A study discovered that urothelial carcinoma has approximately 17 times more PpIX than normal cells^59^. Under certain wavelengths of light (375–440 nm), PpIX in tumor cells changes into an excited fluorescent molecule, producing red fluorescence, and providing a higher contrast between malignant and benign tissues. Currently, HAL is approved for clinical use in the United States and Europe. However, HAL is cytotoxic and can only be administered through bladder perfusion, whereas 5-ALA, a natural amino acid produced by the mitochondria of animals and plants with low toxicity, can be administered orally^60^. In 2017, Japan approved the use of oral 5-ALA as a diagnostic agent. Compared with FC, NBI is simpler, and no special preoperative preparation is required. During the operation, the free switch between NBI and WLC can be achieved by pressing the button.

A more detailed meta-analysis was performed based on the follow-up time, type of photosensitizer, and tumor stage. The results revealed that compared with WLC, 5-ALA, HAL, and NBI provide more advantages. However, 5-ALA revealed no statistical difference in the intermediate-term recurrence rate and detection rate of T1 tumors. Overall, all three methods were recommended. Considering the composition of patients in the original study, the advantages of the new assistive technology are mainly observed in patients with nonmuscle-invasive bladder cancer. Presently, no RCTs have directly compared these three methods. Some studies suggest that HAL is more effective because it is lipophilic compared with 5-ALA, provides deeper tissue penetration, and has higher tumor selectivity, resulting in brighter fluorescence. In addition, the duration of administration before cystoscopy is shorter (1 h vs. 2 h)^49,61^. To explore which method is the most advantageous, we conducted an NMA to indirectly compare the efficacy of the three methods. The results revealed no significant difference between the three methods regarding recurrence and detection rates. In the SUCRA ranking, 5-ALA ranked highest for reducing short-term and long-term recurrence rates. NBI had the highest in intermediate-term recurrence rate. HAL ranked the highest in Ta and T1 bladder cancer detection rates. In CIS, 5-ALA was the highest.

Previous studies have revealed conflicting results regarding the efficacy of these new assistive technologies in reducing the recurrence rate of bladder cancer. Some scholars believe that the recurrence rate between WLC and new assistive technologies is similar because the small-volume lesions missed by WLC can be successfully treated by intravesical chemotherapy or bacillus Calmette-Guérin (BCG) immunotherapy^19^. In contrast, some argue that new assistive technologies provide a more accurate view of tumor margins, enabling adequate resection. In the studies by Geavlete and Ma, NBI observed pathologically confirmed additional extended positive tumor margins in the normal-looking mucosa surrounding the tumor^24,26^. In addition, in the subgroup analysis of the second TURBT, some results were changed. It seems that the advantages of FC and NBI can be weakened to a certain extent by a second TURBT, which also highlights the importance of a second TURBT. However, this subgroup analysis contained less literature and needs further verification. Owing to the limited data provided by the original study, we did not conduct a more detailed subgroup analysis in this study. In some studies, FC was found to have a lower recurrence rate in patients with multiple and recurrent tumors, whereas the advantage was not obvious in patients with single tumors^7,8,15–17^. Similarly, NBI is more advantageous in patients with low-risk, multiple tumors^24,27^. Therefore, whether new assistive technologies are appropriate for all nonmuscular bladder cancer patients or only for subgroups of patients with specific risk characteristics (for example, recurrent, multifocal, and CIS) remains inconclusive.

Regarding disease diagnosis, an ideal detection method should have both sensitivity and specificity of 100%, which is difficult to achieve in the practical application where an increase in sensitivity will lead to a reduction in specificity, and vice versa. This also exists with 5-ALA, HAL, and NBI, which are more sensitive to bladder cancer than WLC; however, the specificity of 5-ALA and HAL is low, and only NBI has the same specificity as WLC. New assistive technologies make it easier to distinguish abnormal tissue from normal tissue, allowing surgeons to find more suspicious areas; however, these suspicious areas also contain a significant proportion of nontumor tissue, such as scars, inflammation, squamous metaplasia, and developmental abnormalities. Many studies indicate that FC and NBI have a higher false-positive rate than WLC^38,44,57^, especially in patients with prior vesical intravesical chemotherapy and TURBT. This may be due to inflammatory changes caused by intravesical treatment. Ronald et al. discovered that the false-positive rate of FC is time-dependent on TURBT, with significantly higher rates of FC false-positives in the first 9 weeks after TURBT (0–9 weeks vs. 9–18weeks: 73 vs. 40%, *P*=0.005), with the lowest rate at 12 weeks^62^. Similarly, the false-positive rate is directly affected by the operatives’ experience^47^. Gkritsios made an interesting point regarding false-positives for FC. He suggested that when the surgeon looks at them at an acute angle, normal thin mucous membranes become overlapping layers of thick cells that produce enhanced fluorescence. This phenomenon is common when a surgeon looks at the bladder neck, trigonometry, or ureteral opening under blue light^22^. This has also been demonstrated in other studies^62,63^. Geavlete pointed out that these areas can be pressed with an electric loop, and if the fluorescence is reduced, it is usually benign and does not need to be excised. Similarly, false-positive biopsies that are common in NBI are considered inflammatory. Previous intravesical chemotherapy increased approximately 20% in the false-positive rate of NBI but did not affect the detection rate^56^. In addition, bleeding interferes with the use of NBI because narrow bands of green and blue light are strongly absorbed by hemoglobin^55^. However, these false-positive tissues are not completely meaningless because some false-positive tissue tests, although considered non-neoplastic lesions, may be precancerous lesions^51^.

Regarding the detection rate, 5-ALA, HAL, and NBI all improved the detection rate of nonmuscle-invasive bladder cancer, and this high detection rate may translate into a low recurrence rate at later stages. Theoretically, complete resection of all bladder tumors is important in preventing their recurrence. In CIS, the high detection rate of new assistive technologies is the most obvious, and the improvement in 5-ALA is the most significant. This is crucial because CIS is relatively easy to ignore during WLC surgery and is a highly malignant tumor with poor differentiation and a high risk of muscular infiltration. Improving the detection rate of CIS for complete excision is important, which is one of the advantages of the new assistive technologies. However, the diagnosis of CIS using new assistive techniques is not perfect, sometimes requiring a random biopsy^44,47^. For example, in Palou’s study, the detection rate of CIS in FC was lower than that in other studies because approximately 50% of the patients in our study underwent a random biopsy, which was higher than that in other studies. As a result, ~26.7% of CIS cases were discovered through a random biopsy^45^. Therefore, a random biopsy may be needed when FC and NBI are used. None of the new assistive technologies showed superior detection rates for muscle-invasive bladder cancer.

Complications were also observed. The main complications of FC reported in the included studies were hematuria, pain, bladder spasm, urinary irritation symptoms, dysuria, and infection. However, most of these complications originate from TURBT and are not strongly associated with photosensitizers. Allergy poisoning symptoms caused by photosensitizers are very rare and are mainly caused by the oral administration of 5-ALA^40,41,52^. FC was generally well-tolerated. No complications of NBI were reported in the included literature.

This study had some limitations. First, in the NMA analysis of recurrence rates, the asymmetric funnel plot revealed a publication bias, which may have affected the final results. Second, the evidence from indirect comparison in NMA is low and cannot replace direct comparison; thus, it requires validation by RCT. Third, few original studies were included in some of the analyses (for example, the T1 tumor detection rate of 5-ALA and the myoinvasive bladder cancer detection rate of NBI). Fourth, due to the limited data provided by the original literature, such as race and sex characteristics, it is difficult for us to conduct relevant subgroup analysis. Whether these potential factors will affect the experimental results needs to be further verified. Fifth, the study’s literature search spanned 32 years, and there are some differences in the technology and treatment methods of the original literature, such as inconsistent types of intravesical chemotherapy drugs used after TURBT, and different models of new assistive technology instruments. In addition, among the literatures that studied the detection rate, even if new assistive technologies are used, the researchers finally identified the suspicious lesions by the naked eyes and then performed excision. Finally, a pathological examination was performed to determine whether the resected specimen was bladder cancer. However, even if FC or NBI are used, there is still the possibility of missing tumors, which may affect the actual results.

## Conclusions

The advantages of new assistive technologies are higher in patients with nonmuscle-invasive bladder cancer. Compared with WLC, 5-ALA, HAL, and NBI reduced the recurrence rate of bladder cancer. Regarding diagnosis, 5-ALA, HAL, and NBI had higher sensitivity but low specificity; only NBI had the same specificity as WLC. The new assistive technology may significantly improve the detection rate of nonmuscle-invasive bladder cancer, especially CIS. However, it is not advantageous for detecting muscle-invasive bladder cancer.

## Ethical approval

Not applicable.

## Sources of funding

Not applicable.

## Author contribution

Z.F., H.S., and J.Y.: study concepts and design; Z.F. and H.S.: drafting of the manuscript; J.L., X.G., B.W., and Y.L.: interpretation of results, revision, and review. Statistical analysis is done by all authors.

## Conflicts of interest disclosure

The authors declare that there are no conflicts of interest.

## Research registration unique identifying number (UIN)

1. Name of the registry: PROSPERO database.
2. Unique identifying number or registration ID: CRD42022327898.
3. Hyperlink to your specific registration (must be publicly accessible and will be checked): https://www.crd.york.ac.uk/PROSPERO/display_record.php?RecordID=327898.

## Guarantor

Junjie Yu.

## Data availability statement

All data generated and analyzed during this study are included in this published article. The data presented in the article may be requested by consulting the correspondence author.

## Provenance and peer review

Not commissioned, externally peer-reviewed.

## Supplementary Material

SUPPLEMENTARY MATERIAL
