# Supplementary figures and images for: Diagnostic and therapeutic effects of fluorescence cystoscopy and narrow-band imaging in bladder cancer: a systematic review and network meta-analysis
Source: Int J Surg. 2023 Aug 1;109(10):3169–77. doi: 10.1097/JS9.0000000000000592 (PMC10583940; doi:10.1097/JS9.0000000000000592)

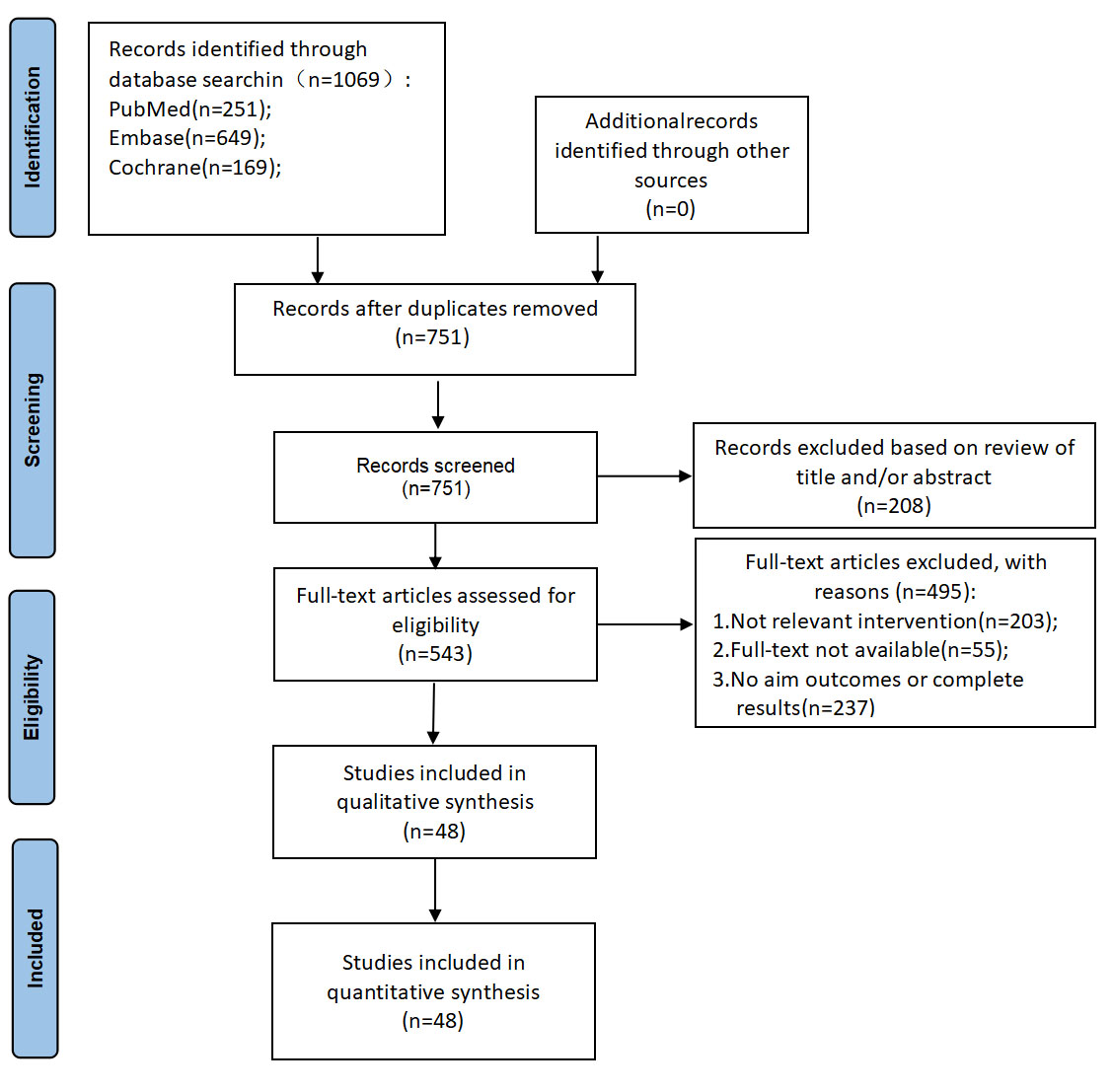

Supplement: SUPPLEMENTARY MATERIAL [file js9-109-3169-s002.jpg]
