# Supplementary material for: Diagnostic and therapeutic effects of fluorescence cystoscopy and narrow-band imaging in bladder cancer: a systematic review and network meta-analysis
Source: Int J Surg. 2023 Aug 1;109(10):3169–77. doi: 10.1097/JS9.0000000000000592 (PMC10583940; doi:10.1097/JS9.0000000000000592)
Supplement: SUPPLEMENTARY MATERIAL [file js9-109-3169-s004.docx]

**Supplemental Online Content**

**sMethods.** Search Strategies

**[Supplementary Fig. 1](https://www.ncbi.nlm.nih.gov/pmc/articles/PMC9913451/#app1-cancers-15-00600).** Flow diagram of the identification process for eligible studies

**[Supplementary Table 1](https://www.ncbi.nlm.nih.gov/pmc/articles/PMC9818995/#app1-cancers-15-00284).** The baseline characteristics of the randomized clinical trials

**[Supplementary Table 2](https://www.ncbi.nlm.nih.gov/pmc/articles/PMC9818995/#app1-cancers-15-00284).** The baseline characteristics of the prospective single-arm studies

**[Supplementary Fig. 2](https://www.ncbi.nlm.nih.gov/pmc/articles/PMC9913451/#app1-cancers-15-00600).** Risk of Bias Summary: Randomized Studies

**[Supplementary Fig. 3](https://www.ncbi.nlm.nih.gov/pmc/articles/PMC9913451/#app1-cancers-15-00600).** Risk of Bias Graph: Randomized Studies

**[Supplementary Fig. 4](https://www.ncbi.nlm.nih.gov/pmc/articles/PMC9913451/#app1-cancers-15-00600).** Methodological quality summary: prospective single-arm studies

**[Supplementary Fig. 5](https://www.ncbi.nlm.nih.gov/pmc/articles/PMC9913451/#app1-cancers-15-00600).** Methodological quality graph: prospective single-arm studies

**[Supplementary Fig. 6](https://www.ncbi.nlm.nih.gov/pmc/articles/PMC9913451/#app1-cancers-15-00600).** Forest plots of short-term recurrence rate of bladder cancer in pairwise meta-analysis

**[Supplementary Fig. 7](https://www.ncbi.nlm.nih.gov/pmc/articles/PMC9913451/#app1-cancers-15-00600).** Forest plots of intermediate-term recurrence rate of bladder cancer in pairwise meta-analysis

**[Supplementary Fig. 8](https://www.ncbi.nlm.nih.gov/pmc/articles/PMC9913451/#app1-cancers-15-00600).** Forest plots of long-term recurrence rate of bladder cancer in pairwise meta-analysis

**[Supplementary Fig. 9](https://www.ncbi.nlm.nih.gov/pmc/articles/PMC9913451/#app1-cancers-15-00600).** Subgroup analysis(intravesical chemotherapy): Forest plots of short-term recurrence rate of bladder cancer in pairwise meta-analysis

**[Supplementary Fig. 1](https://www.ncbi.nlm.nih.gov/pmc/articles/PMC9913451/#app1-cancers-15-00600)0.** Subgroup analysis(intravesical chemotherapy): Forest plots of intermediate-term recurrence rate of bladder cancer in pairwise meta-analysis

**[Supplementary Fig. 1](https://www.ncbi.nlm.nih.gov/pmc/articles/PMC9913451/#app1-cancers-15-00600)1.** Subgroup analysis(intravesical chemotherapy): Forest plots of long-term recurrence rate of bladder cancer in pairwise meta-analysis

**[Supplementary Fig. 1](https://www.ncbi.nlm.nih.gov/pmc/articles/PMC9913451/#app1-cancers-15-00600)2.** Subgroup analysis(second TURBT): Forest plots of short-term recurrence rate of bladder cancer in pairwise meta-analysis

**[Supplementary Fig. 1](https://www.ncbi.nlm.nih.gov/pmc/articles/PMC9913451/#app1-cancers-15-00600)3.** Subgroup analysis(second TURBT): Forest plots of intermediate-term recurrence rate of bladder cancer in pairwise meta-analysis

**[Supplementary Fig. 1](https://www.ncbi.nlm.nih.gov/pmc/articles/PMC9913451/#app1-cancers-15-00600)4.** Subgroup analysis(second TURBT): Forest plots of long-term recurrence rate of bladder cancer in pairwise meta-analysis

**[Supplementary Fig. 1](https://www.ncbi.nlm.nih.gov/pmc/articles/PMC9913451/#app1-cancers-15-00600)5.** The area under the SROC curve

**[Supplementary Fig. 1](https://www.ncbi.nlm.nih.gov/pmc/articles/PMC9913451/#app1-cancers-15-00600)6.** Forest plots of detection rate of Ta stage bladder tumors in pairwise meta-analysis

**[Supplementary Fig. 1](https://www.ncbi.nlm.nih.gov/pmc/articles/PMC9913451/#app1-cancers-15-00600)7.** Forest plots of detection rate of T1 stage bladder tumors in pairwise meta-analysis

**[Supplementary Fig. 1](https://www.ncbi.nlm.nih.gov/pmc/articles/PMC9913451/#app1-cancers-15-00600)8.** Forest plots of detection rate of CIS in pairwise meta-analysis

**[Supplementary Fig. 1](https://www.ncbi.nlm.nih.gov/pmc/articles/PMC9913451/#app1-cancers-15-00600)9.** Forest plots of detection rate of muscle-invasive bladder cancer in pairwise meta-analysis

**[Supplementary Fig. 2](https://www.ncbi.nlm.nih.gov/pmc/articles/PMC9913451/#app1-cancers-15-00600)0.** Forest plots of short-term progression rate of bladder cancer in pairwise meta-analysis

**[Supplementary Fig. 2](https://www.ncbi.nlm.nih.gov/pmc/articles/PMC9913451/#app1-cancers-15-00600)1.** Forest plots of long-term progression rate of bladder cancer in pairwise meta-analysis

**[Supplementary Fig. 22](https://www.ncbi.nlm.nih.gov/pmc/articles/PMC9913451/#app1-cancers-15-00600).** Funnel plots of recurrence rate of bladder cancer in network meta-analysis

**[Supplementary Fig. 23](https://www.ncbi.nlm.nih.gov/pmc/articles/PMC9913451/#app1-cancers-15-00600).** Funnel plots of detection rate of bladder cancer in network meta-analysis

**[Supplementary Fig. 2](https://www.ncbi.nlm.nih.gov/pmc/articles/PMC9913451/#app1-cancers-15-00600)4.** Funnel plots of detection rate of bladder cancer in pairwise meta-analysis

**sMethods.** Search Strategies

Pubmed n=251

#1: (Neoplasm, Urinary Bladder[Title/Abstract]) OR (Urinary Bladder Neoplasm[Title/Abstract]) OR (Neoplasms, Bladder[Title/Abstract]) OR (Bladder Neoplasms[Title/Abstract]) OR (Bladder Neoplasm[Title/Abstract]) OR (Neoplasm, Bladder[Title/Abstract]) OR (Bladder Tumors[Title/Abstract]) OR (Bladder Tumor[Title/Abstract]) OR (Tumor, Bladder[Title/Abstract]) OR (Tumors, Bladder[Title/Abstract]) OR (Urinary Bladder Cancer[Title/Abstract]) OR (Cancer, Urinary Bladder[Title/Abstract]) OR (Malignant Tumor of Urinary Bladder[Title/Abstract]) OR (Cancer of the Bladder[Title/Abstract]) OR (Bladder Cancer[Title/Abstract]) OR (Bladder Cancers[Title/Abstract]) OR (Cancer, Bladder[Title/Abstract]) OR (Cancer of Bladder[Title/Abstract]) OR ("Urinary Bladder Neoplasms"[Mesh])

#2: (NBI[Title/Abstract]) OR ((Band Imaging, Narrow[Title/Abstract]) OR (Band Imagings, Narrow[Title/Abstract]) OR (Imaging, Narrow Band[Title/Abstract]) OR (Imagings, Narrow Band[Title/Abstract]) OR (Narrow Band Imagings[Title/Abstract]) OR (Narrowband Imaging[Title/Abstract]) OR (Imaging, Narrowband[Title/Abstract]) OR (Imagings, Narrowband[Title/Abstract]) OR (Narrowband Imagings[Title/Abstract]) OR ("Narrow Band Imaging"[Mesh])

#3: from 1990/1/1 - 2022/4/1

#4: #1 AND #2 AND #3

#5: (Acid, Aminolevulinic[Title/Abstract]) OR (Delta-Aminolevulinic Acid[Title/Abstract]) OR (Acid, Delta-Aminolevulinic[Title/Abstract]) OR (Delta Aminolevulinic Acid[Title/Abstract]) OR (Levulan[Title/Abstract]) OR (5-Aminolaevulinate[Title/Abstract]) OR (5 Aminolaevulinate[Title/Abstract]) OR (5-Aminolevulinate[Title/Abstract]) OR (5 Aminolevulinate[Title/Abstract]) OR (Aminolevulinic Acid Hydrochloride[Title/Abstract]) OR (Acid Hydrochloride, Aminolevulinic[Title/Abstract]) OR (Hydrochloride, Aminolevulinic Acid[Title/Abstract]) OR ("Aminolevulinic Acid"[Mesh])

#6: (hexaminolevulinate[Title/Abstract]) OR (5-aminolevulinate[Title/Abstract]) OR (5-ALA[Title/Abstract]) OR (HAL[Title/Abstract]) OR (photodynamic diagnosis[Title/Abstract]) OR (PDD[Title/Abstract]) OR (fluorescence cystoscopy[Title/Abstract]) OR (blue light lystoscopy[Title/Abstract]) OR (fluorescence[Title/Abstract])

#7: ("Clinical Trial" [Publication Type])

#8: #5 OR #6

#9: #8 AND #1 AND #7 AND #3

#10: #9 OR #4

Cochrane n=169

#1: MeSH descriptor: [Urinary Bladder Neoplasms] explode all trees

#2: (Neoplasm, Urinary Bladder):ab,ti,kw OR (Urinary Bladder Neoplasm):ab,ti,kw OR (Neoplasms, Bladder):ab,ti,kw OR (Bladder Neoplasms):ab,ti,kw OR (Bladder Neoplasm):ab,ti,kw OR (Neoplasm, Bladder):ab,ti,kw OR (Bladder Tumors):ab,ti,kw OR (Bladder Tumor):ab,ti,kw OR (Tumor, Bladder):ab,ti,kw OR (Tumors, Bladder):ab,ti,kw OR (Urinary Bladder Cancer):ab,ti,kw OR (Cancer, Urinary Bladder):ab,ti,kw OR (Malignant Tumor of Urinary Bladder):ab,ti,kw OR (Cancer of the Bladder):ab,ti,kw OR (Bladder Cancer):ab,ti,kw OR (Bladder Cancers):ab,ti,kw OR (Cancer, Bladder):ab,ti,kw OR (Cancer of Bladder):ab,ti,kw

#3: MeSH descriptor: [Narrow Band Imaging] explode all trees

#4: (NBI):ab,ti,kw OR (Band Imaging, Narrow):ab,ti,kw OR (Band Imagings, Narrow):ab,ti,kw OR (Imaging, Narrow Band):ab,ti,kw OR (Imagings, Narrow Band):ab,ti,kw OR (Narrow Band Imagings):ab,ti,kw OR (Narrowband Imaging):ab,ti,kw OR (Imaging, Narrowband):ab,ti,kw OR (Imagings, Narrowband):ab,ti,kw OR (Narrowband Imagings):ab,ti,kw

#5: #1 OR #2

#6: #3 OR #4

#7: #5 AND #6

#8: MeSH descriptor: [Aminolevulinic Acid] explode all trees

#9: (Acid, Aminolevulinic):ab,ti,kw OR (Delta-Aminolevulinic Acid):ab,ti,kw OR (Acid, Delta-Aminolevulinic):ab,ti,kw OR (Delta Aminolevulinic Acid):ab,ti,kw OR (Levulan):ab,ti,kw OR (Aminolaevulinate):ab,ti,kw OR (5 Aminolaevulinate):ab,ti,kw OR (Aminolevulinate):ab,ti,kw OR (5 Aminolevulinate):ab,ti,kw OR (Aminolevulinic Acid Hydrochloride):ab,ti,kw OR (Acid Hydrochloride, Aminolevulinic):ab,ti,kw OR (Hydrochloride, Aminolevulinic Acid):ab,ti,kw

#10: (Photodynamic diagnosis):ab,ti,kw OR (PDD):ab,ti,kw OR (fluorescence):ab,ti,kw OR (fluorescence diagnosis):ab,ti,kw OR (hexaminolevulinate):ab,ti,kw OR (aminolevulinate):ab,ti,kw OR (ALA):ab,ti,kw OR (HAL):ab,ti,kw OR (blue light lystoscopy):ab,ti,kw

#11: #8 OR #9 OR #10

#12: #11 AND #5

#13: #7 OR #12

EMBASE n=649

#1: 'bladder tumor'/exp

#2: 'neoplasm, urinary bladder':ab,ti,kw OR 'urinary bladder neoplasm':ab,ti,kw OR 'neoplasms, bladder':ab,ti,kw OR 'bladder neoplasms':ab,ti,kw OR 'bladder neoplasm':ab,ti,kw OR 'neoplasm, bladder':ab,ti,kw OR 'bladder tumors':ab,ti,kw OR 'bladder tumor':ab,ti,kw OR 'tumor, bladder':ab,ti,kw OR 'tumors, bladder':ab,ti,kw OR 'urinary bladder cancer':ab,ti,kw OR 'cancer, urinary bladder':ab,ti,kw OR 'malignant tumor of urinary bladder':ab,ti,kw OR 'cancer of the bladder':ab,ti,kw OR 'bladder cancer':ab,ti,kw OR 'bladder cancers':ab,ti,kw OR 'cancer, bladder':ab,ti,kw OR 'cancer of bladder':ab,ti,kw

#3: 'narrow band imaging'/exp

#4: 'nbi':ab,ti,kw OR 'band imaging, narrow':ab,ti,kw OR 'band imagings, narrow':ab,ti,kw OR 'imaging, narrow band':ab,ti,kw OR 'imagings, narrow band':ab,ti,kw OR 'narrow band imagings':ab,ti,kw OR 'narrowband imaging':ab,ti,kw OR 'imaging, narrowband':ab,ti,kw OR 'imagings, narrowband':ab,ti,kw OR 'narrowband imagings':ab,ti,kw

#5: #1 OR #2

#6: #3 OR #4

#7: #5 AND #6

#8: 'aminolevulinic acid'/exp

#9: 'acid, aminolevulinic':ab,ti,kw OR 'delta-aminolevulinic acid':ab,ti,kw OR 'acid, delta-aminolevulinic':ab,ti,kw OR 'delta aminolevulinic acid':ab,ti,kw OR 'levulan':ab,ti,kw OR '5-aminolaevulinate':ab,ti,kw OR '5 aminolaevulinate':ab,ti,kw OR '5-aminolevulinate':ab,ti,kw OR '5 aminolevulinate':ab,ti,kw OR 'aminolevulinic acid hydrochloride':ab,ti,kw OR 'acid hydrochloride, aminolevulinic':ab,ti,kw OR 'hydrochloride, aminolevulinic acid':ab,ti,kw

#10: 'photodynamic diagnosis':ab,ti,kw OR 'pdd':ab,ti,kw OR 'fluorescence':ab,ti,kw OR 'fluorescence diagnosis':ab,ti,kw OR 'hexaminolevulinate':ab,ti,kw OR '5-aminolevulinate':ab,ti,kw OR '5-ala':ab,ti,kw OR 'hal':ab,ti,kw OR 'blue light lystoscopy':ab,ti,kw

#11: #8 OR #9 OR #10

#12: #5 AND #11

#13: 'clinical trial'/exp

#14: #12 AND #13

#15: #7 OR #14

**[Supplementary Fig. 1](https://www.ncbi.nlm.nih.gov/pmc/articles/PMC9913451/#app1-cancers-15-00600).** Flow diagram of the identification process for eligible studies.

**
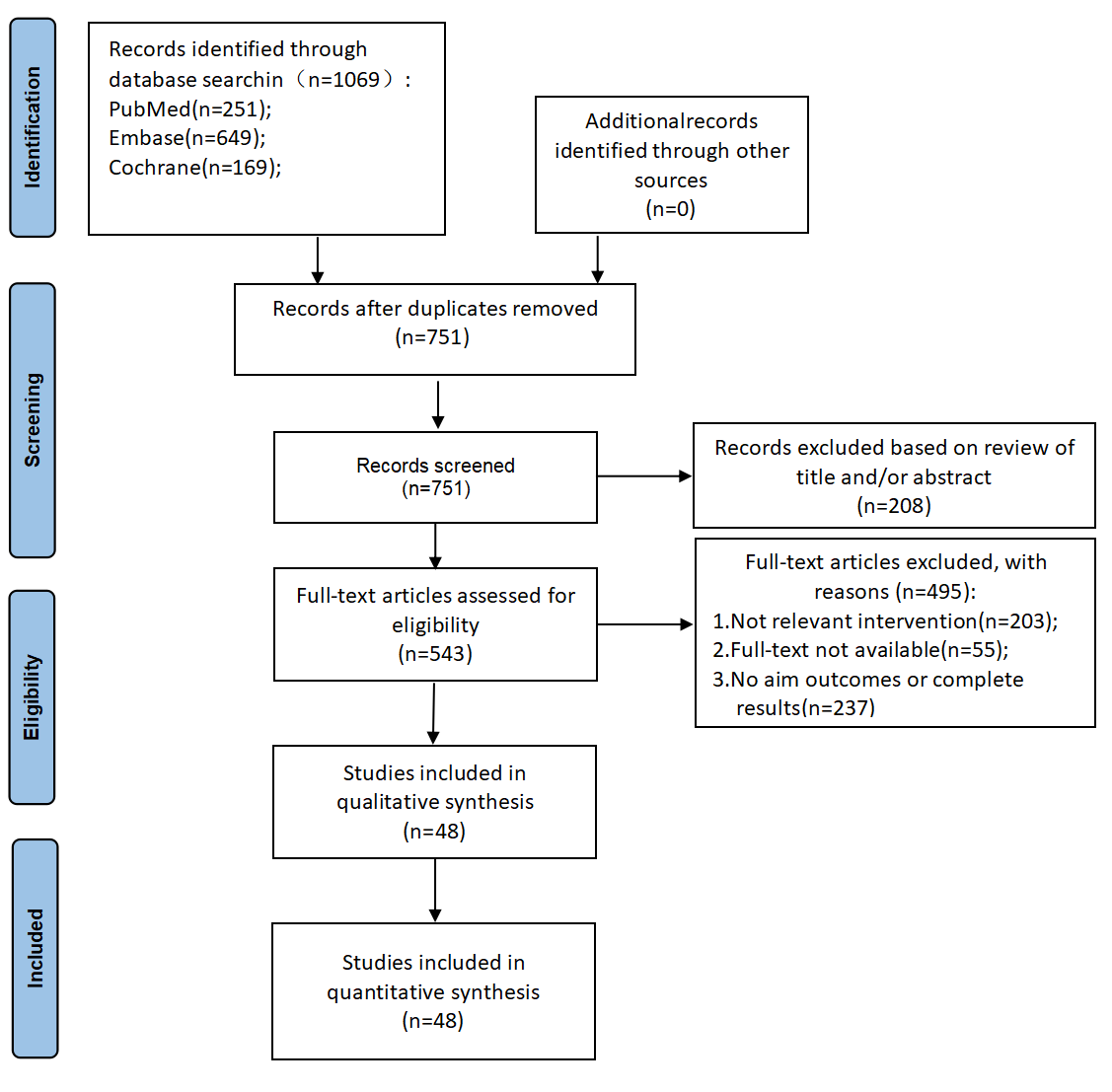
**

| **[Supplementary Table 1](https://www.ncbi.nlm.nih.gov/pmc/articles/PMC9818995/#app1-cancers-15-00284).** The baseline characteristics of the randomized clinical trials.  RCT: randomized clinical trials. 5-ALA: 5-aminolevulinic acid; HAL: hexaminolaevu-linate; NBI:narrow band imaging; WIC:white light cystoscopy. | | | | | | | |
| --- | --- | --- | --- | --- | --- | --- | --- |
| **Study** | **Design** | **Intervention** | **Follow-up** | **Outcome** | **Country** | **Gender(M/F)** | **age** |
| [C R Riedl2001](https://pubmed.ncbi.nlm.nih.gov/?sort=pubdate&term=Riedl+CR&cauthor_id=11257651) | RCT | WLC:51 | 60M | Recurrence rate:WLC:74.51% ALA:58.82% | multicenter/Austria | WLC:37/14 | WLC:67 (19-86) |
|  |  | ALA:51 |  | Progression rate:WLC:17.65% ALA:7.84% |  | ALA:36/15 | ALA:70 (54-89) |
| Marko Babjuk2005 | RCT | WLC:62 | 24M | Recurrence rate:WLC:72.58% ALA:60% | Single center/Czech Republic | WLC:39/23 | WLC:69.8 |
|  |  | ALA:60 |  | Progression rate:WLC:8.06% ALA:8.33% |  | ALA:43/17 | ALA:67.9 |
| Martin Kriegmair2002 | RCT | WLC:64 | 2W | Recurrence rate:WLC:59.38% ALA:38.46% | Multicenter/Germany Austria | WLC:45/19 | WLC:69.6 (34–94) |
|  |  | ALA:65 |  |  |  | ALA:53/12 | ALA:69.3 (38–88) |
| [Thomas Filbeck2002](https://pubmed.ncbi.nlm.nih.gov/?sort=pubdate&term=Filbeck+T&cauthor_id=12050494) | RCT | WLC:103 | 83M(median) | Recurrence rate:WLC:41.75% ALA:20.45% | Single center/Germany | WLC:78/25 | WLC:70 (32–89) |
|  |  | ALA:88 |  | Progression rate:WLC:12% ALA:19.05% |  | ALA:65/23 | ALA:68(31–88) |
| Arnulf Stenzl2011 | RCT | WLC:176 | 12M | Recurrence rate:WLC:25.57% ALA:31.15% | Multicenter/Germany Austria | 259/100 |  |
|  |  | ALA:183 |  | Progression rate:WLC:8.52% ALA:7.65% |  |  |  |
| Martin C Schumacher2010 | RCT | WLC:138 | 12M | Recurrence rate:WLC:44.2% ALA:44.68% | Multicenter/Sweden | WLC:104/34 | WLC:68.9±10.8 |
|  |  | ALA:141 |  | Progression rate:WLC:10.77% ALA:8.82% |  | ALA:103/38 | ALA:70.1±10.1 |
| Alexander I Rolevich2017 | RCT | WLC:203 | WLC:65M(median) | Recurrence rate:WLC:42.86% ALA:31.61% | Single center/Republic of Belarus | WLC:156/47 | 66 (18–87) |
|  |  | ALA:174 | ALA:54M(median) | Progression rate:WLC:9.36% ALA:4.02% |  | ALA:134/40 |  |
| O Drăgoescu2011 | RCT | WLC:22 | 12M | Recurrence rate:WLC:45.45% HAL:18.18% | Single center/Romania |  | WLC:62.09±12.46 |
|  |  | HAL:22 |  | Progression rate:WLC:9.09% HAL:4.55% |  |  | HAL:58.77±14.31 |
| Arnulf Stenzl2010 | RCT | WLC:280 | 53M(median) | Recurrence rate:WLC:68.2% HAL:61.96% | Multicenter/USA Canada Europe | WLC:223/57 | WLC:69.6 ±10.7 |
|  |  | HAL:271 |  | Progression rate:WLC:2.5% HAL:1.85% |  | HAL:212/59 | HAL:68.0 ±10.8 |
| Bogdan Geavlete2012 | RCT | WLC:181 | 24M | Recurrence rate:WLC:36.11% HAL:31.2% | Single center/Romania | 264/95 |  |
|  |  | HAL:181 |  | Progression rate:WLC:7.02% HAL:4% |  |  |  |
| Theocharis Karaolides2012 | RCT | WLC:45 | WLC:4.5-25M | Recurrence rate:WLC:40% HAL:17.07% | Single center/Greece | WLC:40/5 | WLC:63.82 (39-88) |
|  |  | HAL:41 | HAL:6-25M | Progression rate:WLC:4.44% HAL:0% |  | HAL:33/8 | HAL:66.29(37-82) |
| Petru Octavian Drăgoescu2017 | RCT | WLC:56 | 60-83M | Recurrence rate:WLC:67.86% HAL:49.12% | Single center/Romania |  | WLC:60.3±10.2 |
|  |  | HAL:57 |  | Progression rate:WLC:10.71% HAL:8.77% |  |  | HAL:59.4±9.9 |
| Timothy O'Brien2013 | RCT | WLC:120 | 12M | Recurrence rate:WLC:35.37% HAL:31.4% | Double center/UK | WLC:88/32 | WLC:68 (29–90) |
|  |  | HAL:129 |  |  |  | HAL:95/34 | HAL:68 (31–95) |
| Yann Neuzillet2014 | RCT | WLC:79 | 6W | Recurrence rate:WLC:52% HAL:46.51% | Double center/France | 133/18 | WLC:74±10.4 |
|  |  | HAL:72 |  |  |  |  | HAL:74±10.3 |
| Gregers G Hermann2011 | RCT | WLC:118 | 12M | Recurrence rate:WLC:47.3% HAL:30.51% | Double center/Denmark |  | WLC:69 (41-92) |
|  |  | HAL:115 |  |  |  |  | HAL:71 (35-96) |
| P Gkritsios2014 | RCT | WLC:50 | 24M | Recurrence rate:WLC:32.43% HAL:20.83% | Single center/Greece | WLC:44/6 | WLC:68.24 |
|  |  | HAL:54 |  |  |  | HAL:43/11 | HAL:66 |
| Ditte Drejer2020 | RCT | WLC:348 | 12M | Recurrence rate:WLC:42.43% HAL:35.03% | Multicenter/Denmark | WLC:247/101 | WLC:69.9 (22-97) |
|  |  | HAL:351 |  |  |  | HAL:252/99 | HAL:70.3 (22-97) |
| Bogdan Geavlete2012 | RCT | WLC:110 | 12M | Recurrence rate:WLC:17.78% NBI:7.87% | Single center/Romania | WLC:82/28 | WLC:64.5(30-83) |
|  |  | NBI:110 |  |  |  | NBI:79/31 | NBI:63.7(31-84) |
| Angelo Naselli2012 | RCT | WLC:72 | 12M | Recurrence rate:WLC:51.39% NBI:31.58% | Double center/Italy | WLC:17/55 | WLC:71.6±12.4 |
|  |  | NBI:76 |  |  |  | NBI:12/64 | NBI:70.8±10.3 |
| Ma Tianjia2015 | RCT | WLC:92 | 12M | Recurrence rate:WLC:38.04% NBI:18.6% | Single center/China | WLC:79/13 | WLC:62±8 |
|  |  | NBI:86 |  |  |  | NBI:69/17 | NBI:63±9 |
| Seiji Naito2016 | RCT | WLC:481 | 12M | Recurrence rate:WLC:27.11% NBI:25.43% | Multicenter | WLC:383/98 | WLC:65.8±12.5 |
|  |  | NBI:484 |  |  |  | NBI:390/94 | NBI:66.7±12.3 |
| Harry W Herr2015 | RCT | WLC:127 | 24M | Recurrence rate:WLC:33.07% NBI:22.05% | Single center/USA | WLC:95/32 | WLC:68(42–99) |
|  |  | NBI:127 |  | Progression rate:WLC:13.39% NBI:6.3% |  | NBI:88/39 | NBI:67(36–93) |
| Buaban K2018 | RCT | WLC:31 | 3M | Recurrence rate:WLC:61.29% NBI:25% | Single center/Thailand | WLC:20/11 | WLC:66 |
|  |  | NBI:44 |  |  |  | NBI:36/8 | NBI:75 |
| Seung Bin Kim2018 | RCT | WLC:67 | 12M | Recurrence rate:WLC:25.71% NBI:12.82% | Single center/South Korea | WLC:54/13 | WLC:66.96±11.51 |
|  |  | NBI:85 |  |  |  | NBI:62/23 | NBI:64.54±12.01 |
| JY Lee2014 | RCT | WLC:35 | 24M | Recurrence rate:WLC:22.86% NBI:15.15% | Single center/South Korea |  | WLC:63.82±12.31 |
|  |  | NBI:33 |  |  |  |  | NBI: 63.03±12.43 |
| S Tschirdewahn2020 | RCT | WLC:70 | 48M | Recurrence rate:WLC:80% NBI:73.08% | Single center/Germany | WLC:59/11 | WLC:75 (53–97) |
|  |  | NBI:78 |  |  |  | NBI:56/22 | NBI:76 (54–92) |

| **[Supplementary Table 2](https://www.ncbi.nlm.nih.gov/pmc/articles/PMC9818995/#app1-cancers-15-00284).** The baseline characteristics of the prospective single-arm studies.  5-ALA: 5-aminolevulinic acid; HAL: hexaminolaevu-linate; NBI:narrow band imaging; WIC:white light cystoscopy. | | | | | | |
| --- | --- | --- | --- | --- | --- | --- |
| **Study** | **Design** | **Intervention** | **Number of Samples** | **Country** | **Gender(M/F)** | **age** |
|  |  |  |  |  |  |  |
| T Filbeck1999 | Prospective | ALA/WLC | 347 | Germany |  |  |
| F Koenig1999 | Prospective | ALA/WLC | 130 | Germany | 11/44 | 66(31-87) |
| Yasushi Nakai2018 | Prospective | ALA/WLC | 513 | multicenter/Japan | 51/9 | 70.1±7.9 |
| Keiji Inoue2016 | Prospective | ALA/WLC | 1284 | multicenter/Japan | 158/34 | 69.1±9.57 |
| S Schneeweiss1999 | Prospective | ALA/WLC | 328 | USA | 170/38 | 64.8±12 |
| Patrice Jichlinski2003 | Prospective | HAL/WLC | 422 | multicenter/Europe | 39/13 | 72±12 |
| Yves Fradet2007 | Prospective | HAL/WLC | 695 | multicenter/USA,Canada | 223/75 | 67±11 |
| Juan Palou2015 | Prospective | HAL/WLC | 1569 | multicenter/Spain | 243/40 | 67.5(42-95) |
| Alberto Lapini2012 | Prospective | HAL/WLC | 234 | multicenter/Italy | 80/16 |  |
| J P Burgués2011 | Prospective | HAL/WLC | 1659 | multicenter/Spain | 270/35 | 66.69(39-93) |
| Dieter Jocham2005 | Prospective | HAL/WLC | 382 | multicenter/Germany | 107/39 | 67(33-91) |
| Jae Seung Lee2012 | Prospective | HAL/WLC | 134 | Korea | 25/5 | 60.43±9.22 |
| Joerg Schmidbauer2009 | Prospective | HAL/WLC | 364 | Austria | 49/17 | 67(38-84) |
| A Ferré2013 | Prospective | HAL/WLC | 368 | France | 89/18 | 66.4(35-97) |
| Siamak Daneshmand2018 | Prospective | HAL/WLC | 1632 | multicenter/USA | 446/87 | 72(23-101) |
| Eleanor R Ray2010 | Prospective | HAL/WLC | 120 | UK | 30/7 | 70(49-82) |
| F Saint2010 | Prospective | HAL/WLC | 92 | France |  |  |
| Zhangqun Ye2015 | Prospective | NBI/WLC | 300 | multicenter/China | 87/24 | 61±10.7 |
| Katsunori Tatsugami2010 | Prospective | NBI/WLC | 313 | multicenter/Japan | 88/16 | 70.6(38-90) |
| Yi-Jun Shen2012 | Prospective | NBI/WLC | 309 | China | 62/16 | 68(33–75) |
| Evelyne C C Cauberg2010 | Prospective | NBI/WLC | 389 | Double center/Netherlands,Czech Republic | 70/25 | 70.6±10.7 |
| Pejman Shadpour2016 | Prospective | NBI/WLC | 199 | Iran | 34/16 | 63.86±10.05 |

**[Supplementary Fig. 2](https://www.ncbi.nlm.nih.gov/pmc/articles/PMC9913451/#app1-cancers-15-00600).** Risk of Bias Summary: Randomized Studies.


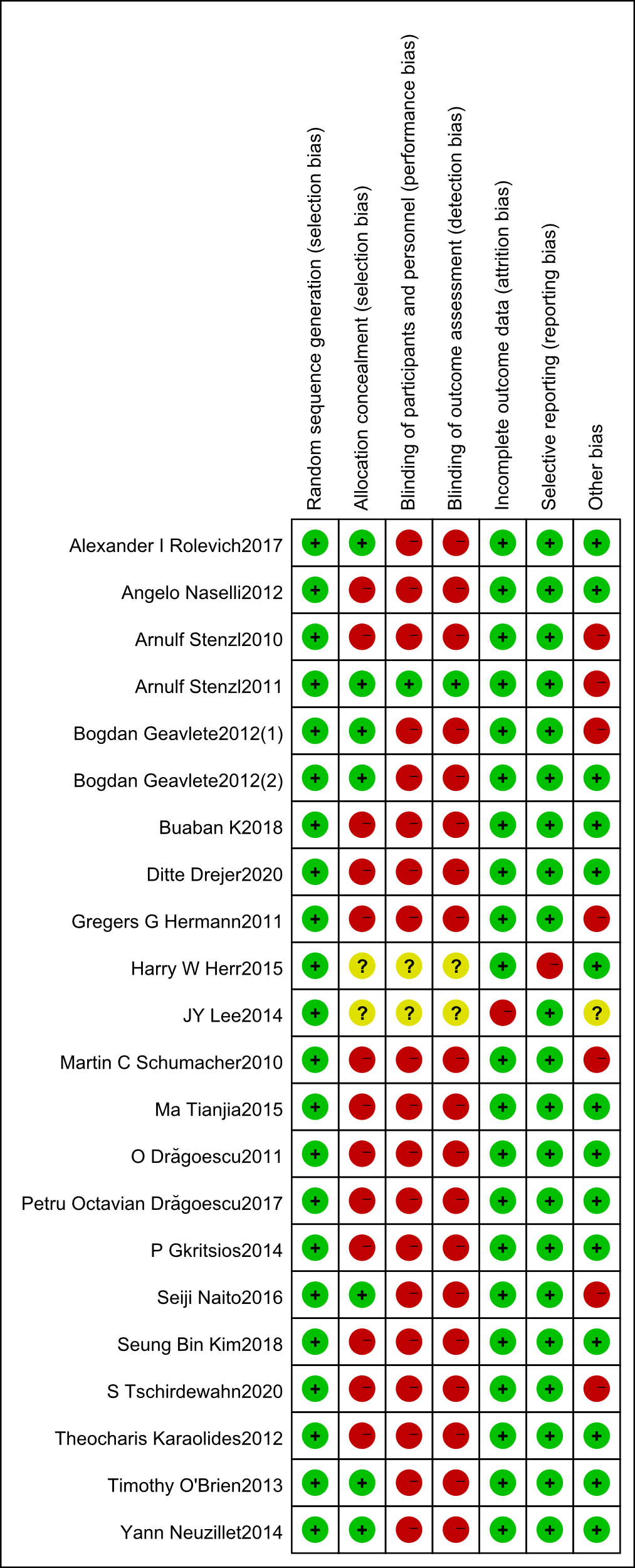


**[Supplementary Fig. 3](https://www.ncbi.nlm.nih.gov/pmc/articles/PMC9913451/#app1-cancers-15-00600).** Risk of Bias Graph: Randomized Studies.


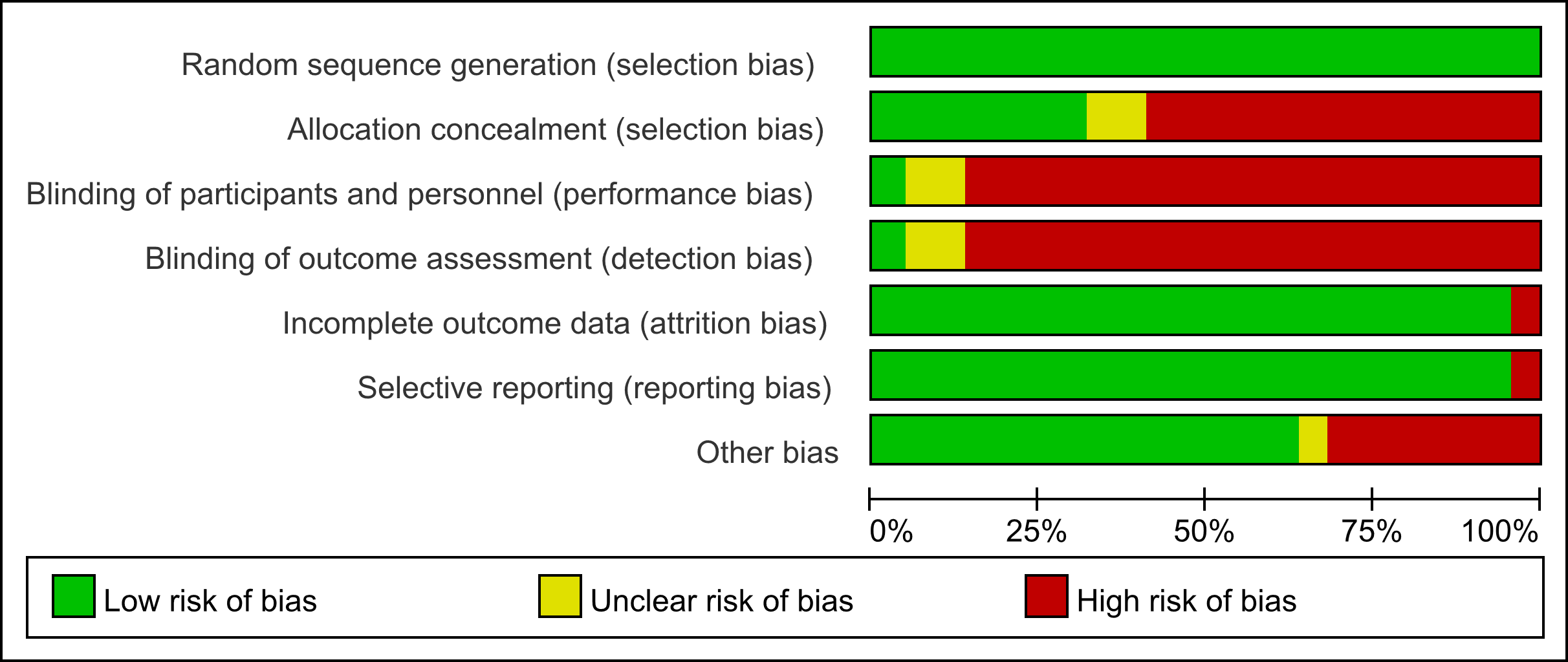


**[Supplementary Fig. 4](https://www.ncbi.nlm.nih.gov/pmc/articles/PMC9913451/#app1-cancers-15-00600).** Methodological quality summary: prospective single-arm studies

**
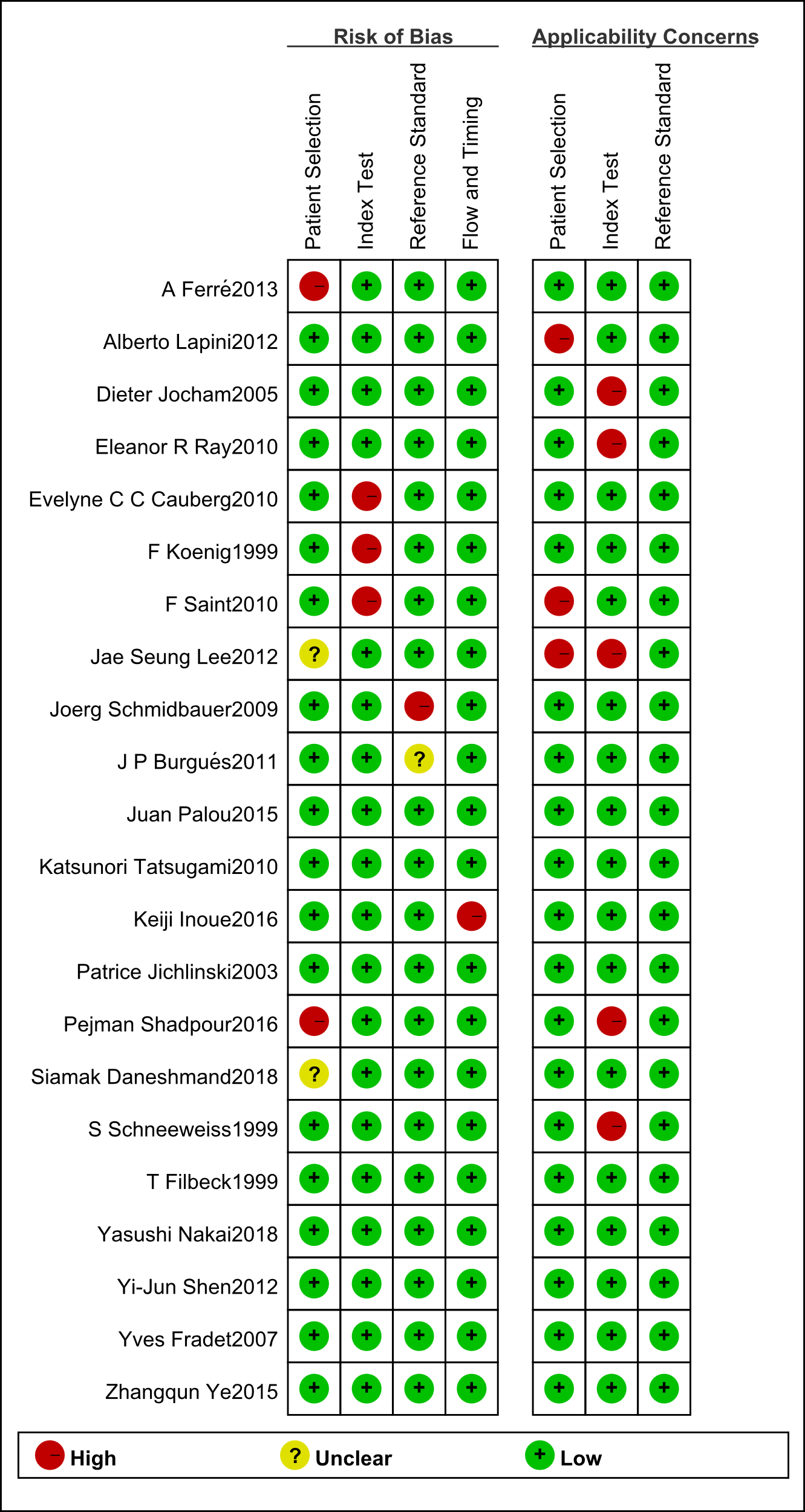
**

**[Supplementary Fig. 5](https://www.ncbi.nlm.nih.gov/pmc/articles/PMC9913451/#app1-cancers-15-00600).** Methodological quality graph: prospective single-arm studies


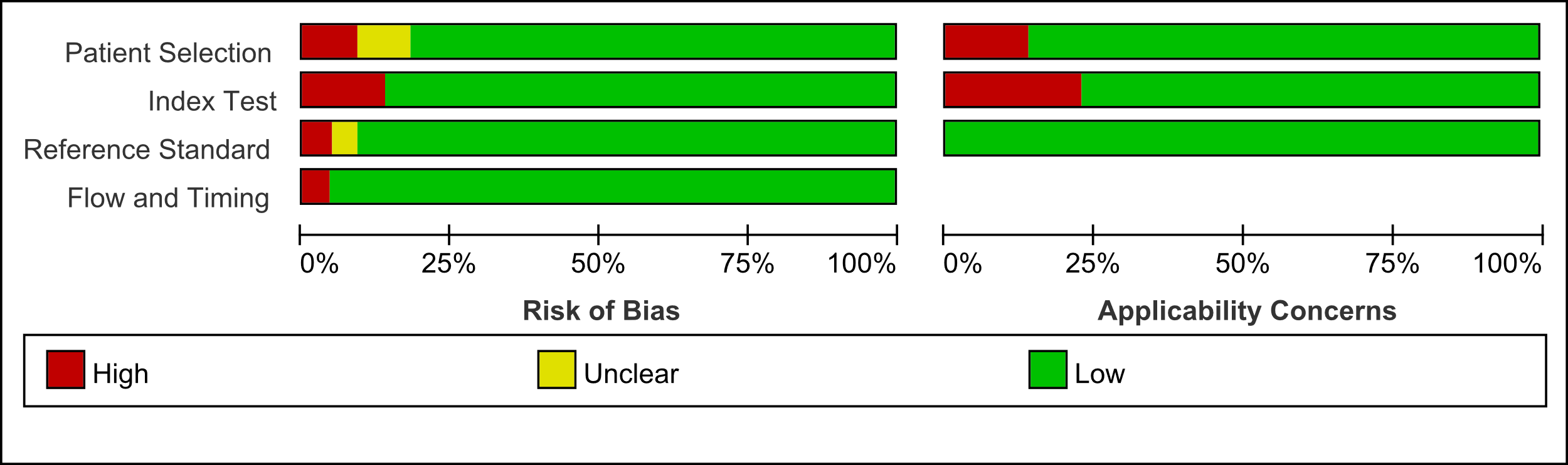


**[Supplementary Fig. 6](https://www.ncbi.nlm.nih.gov/pmc/articles/PMC9913451/#app1-cancers-15-00600).** Forest plots of short-term recurrence rate of bladder cancer in pairwise meta-analysis. (A) 5-ALA VS WLC, (B) HAL VS WLC, (C) NBI VS WLC. 5-ALA: 5-aminolevulinic acid; HAL: hexaminolaevu-linate; NBI:narrow band imaging; WIC:white light cystoscopy.


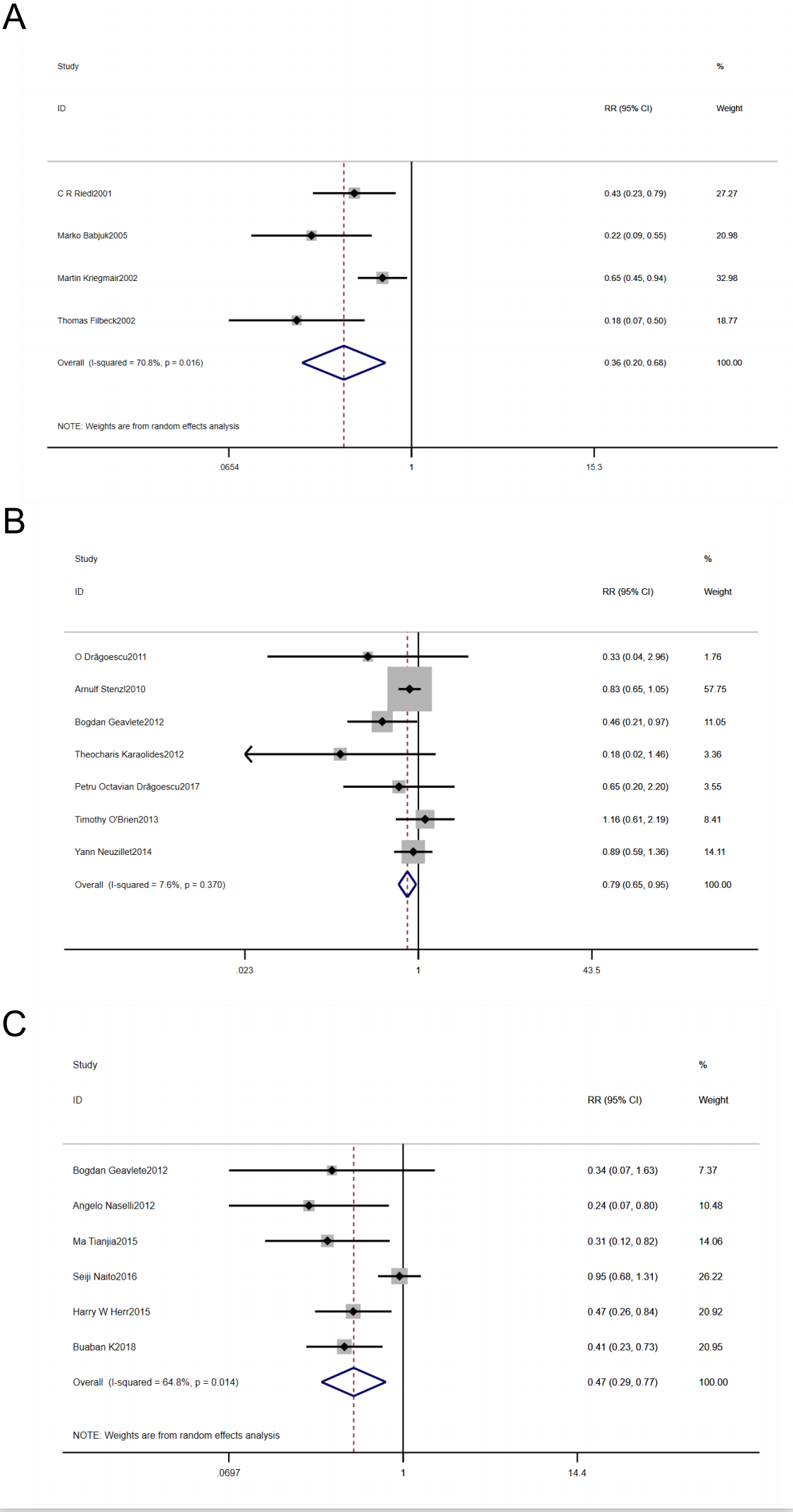


**[Supplementary Fig. 7](https://www.ncbi.nlm.nih.gov/pmc/articles/PMC9913451/#app1-cancers-15-00600).** Forest plots of intermediate-term recurrence rate of bladder cancer in pairwise meta-analysis. (A) 5-ALA VS WLC, (B) HAL VS WLC, (C) NBI VS WLC. 5-ALA: 5-aminolevulinic acid; HAL: hexaminolaevu-linate; NBI:narrow band imaging; WIC:white light cystoscopy.


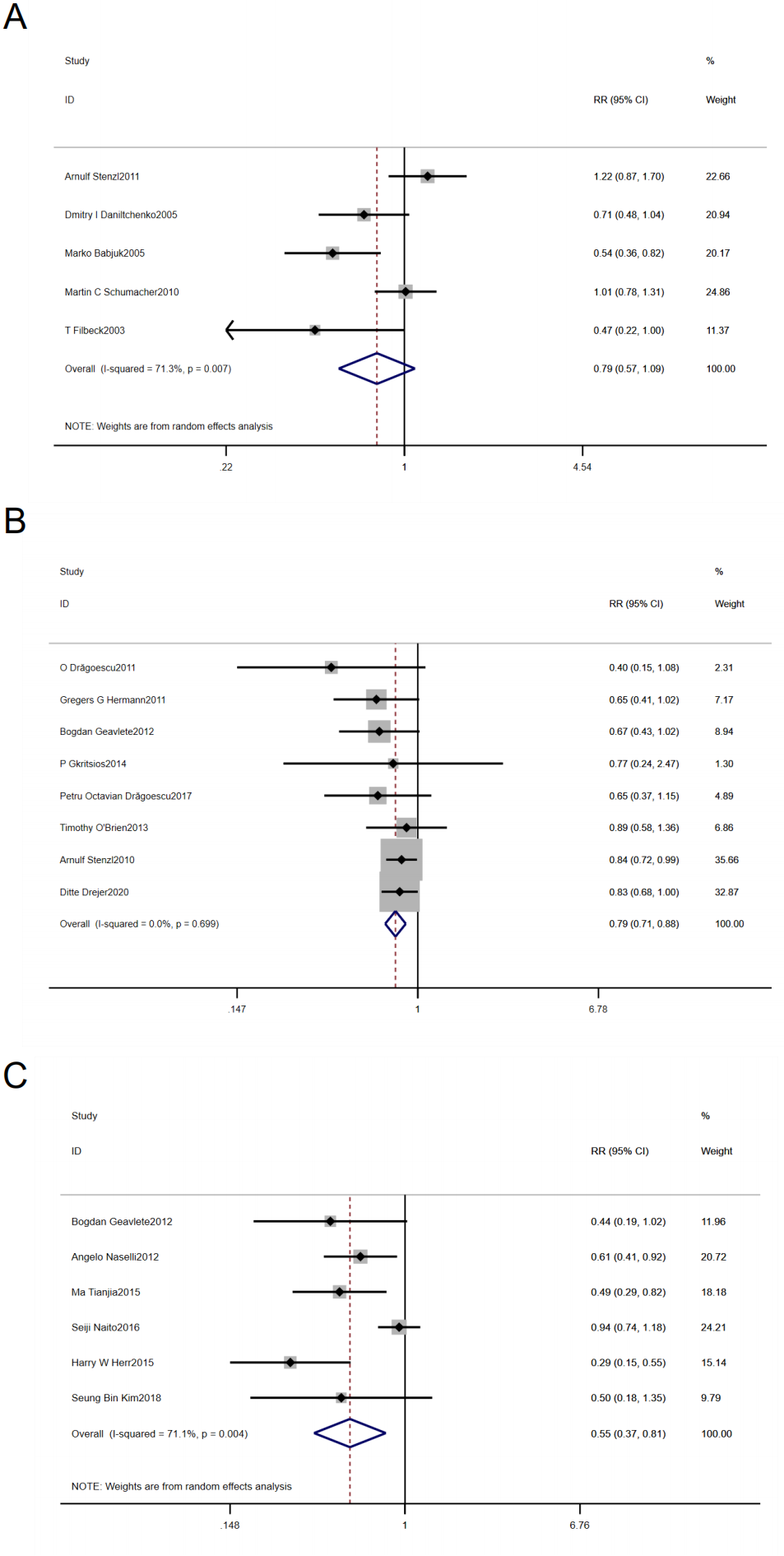


**[Supplementary Fig. 8](https://www.ncbi.nlm.nih.gov/pmc/articles/PMC9913451/#app1-cancers-15-00600).** Forest plots of long-term recurrence rate of bladder cancer in pairwise meta-analysis. (A) 5-ALA VS WLC, (B) HAL VS WLC, (C) NBI VS WLC. 5-ALA: 5-aminolevulinic acid; HAL: hexaminolaevu-linate; NBI:narrow band imaging; WIC:white light cystoscopy.


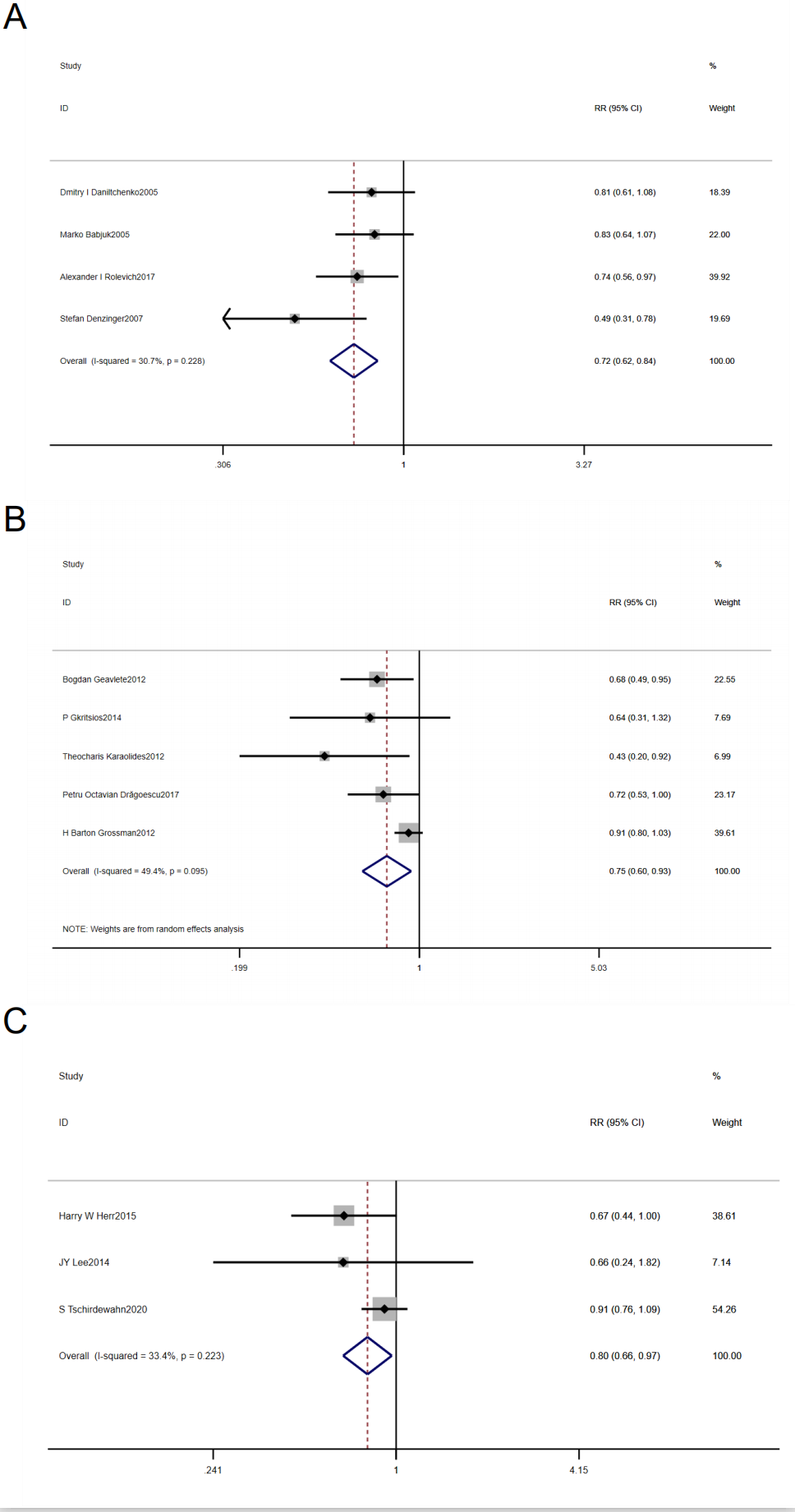


**[Supplementary Fig. 9](https://www.ncbi.nlm.nih.gov/pmc/articles/PMC9913451/#app1-cancers-15-00600).** Subgroup analysis(intravesical chemotherapy): Forest plots of short-term recurrence rate of bladder cancer in pairwise meta-analysis. (A) 5-ALA VS WLC, (B) HAL VS WLC, (C) NBI VS WLC. 5-ALA: 5-aminolevulinic acid; HAL: hexaminolaevu-linate; NBI:narrow band imaging; WIC:white light cystoscopy.

**
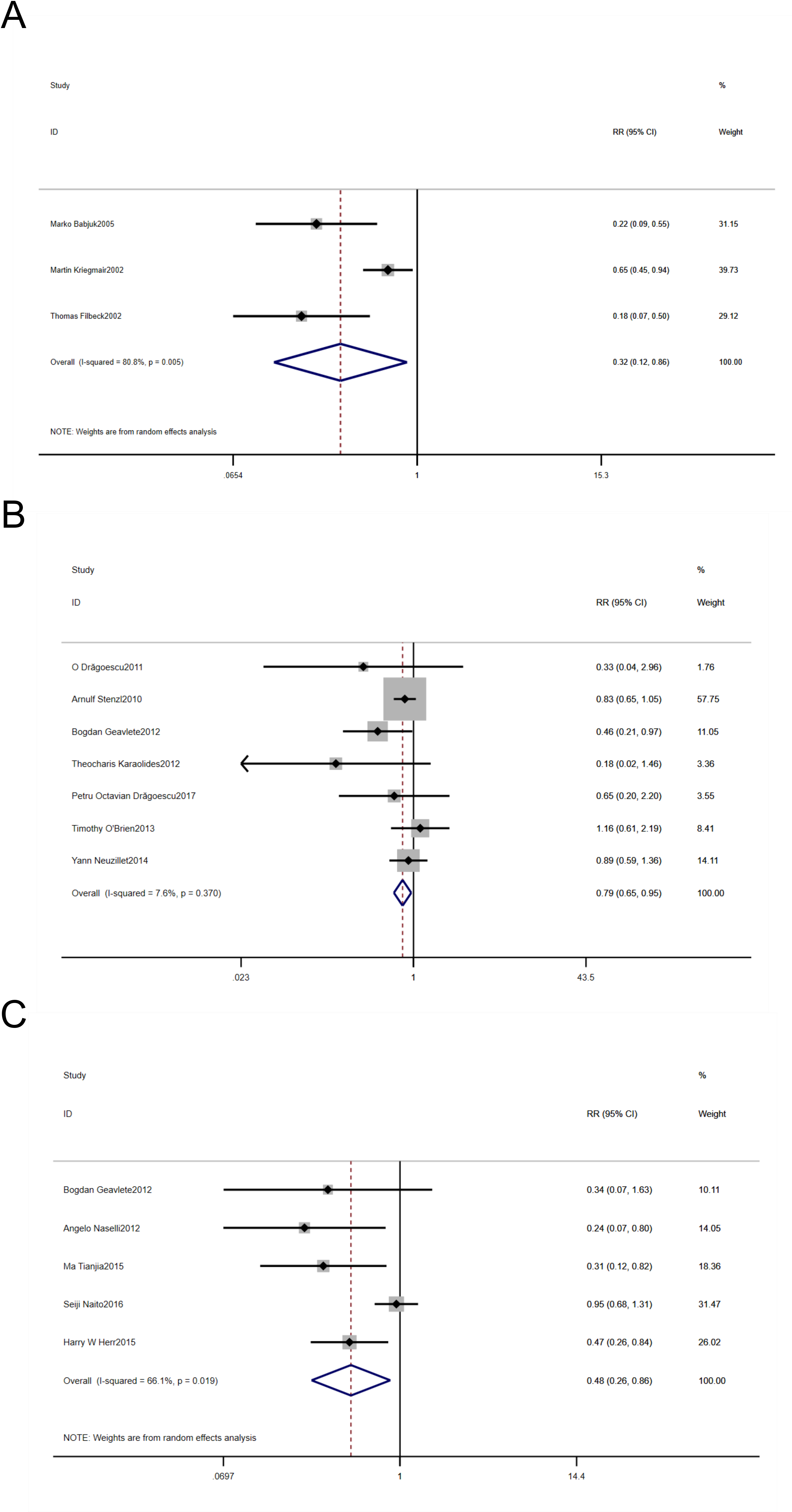
**

**[Supplementary Fig. 1](https://www.ncbi.nlm.nih.gov/pmc/articles/PMC9913451/#app1-cancers-15-00600)0.** Subgroup analysis(intravesical chemotherapy): Forest plots of intermediate-term recurrence rate of bladder cancer in pairwise meta-analysis. (A) 5-ALA VS WLC, (B) HAL VS WLC, (C) NBI VS WLC. 5-ALA: 5-aminolevulinic acid; HAL: hexaminolaevu-linate; NBI:narrow band imaging; WIC:white light cystoscopy.**
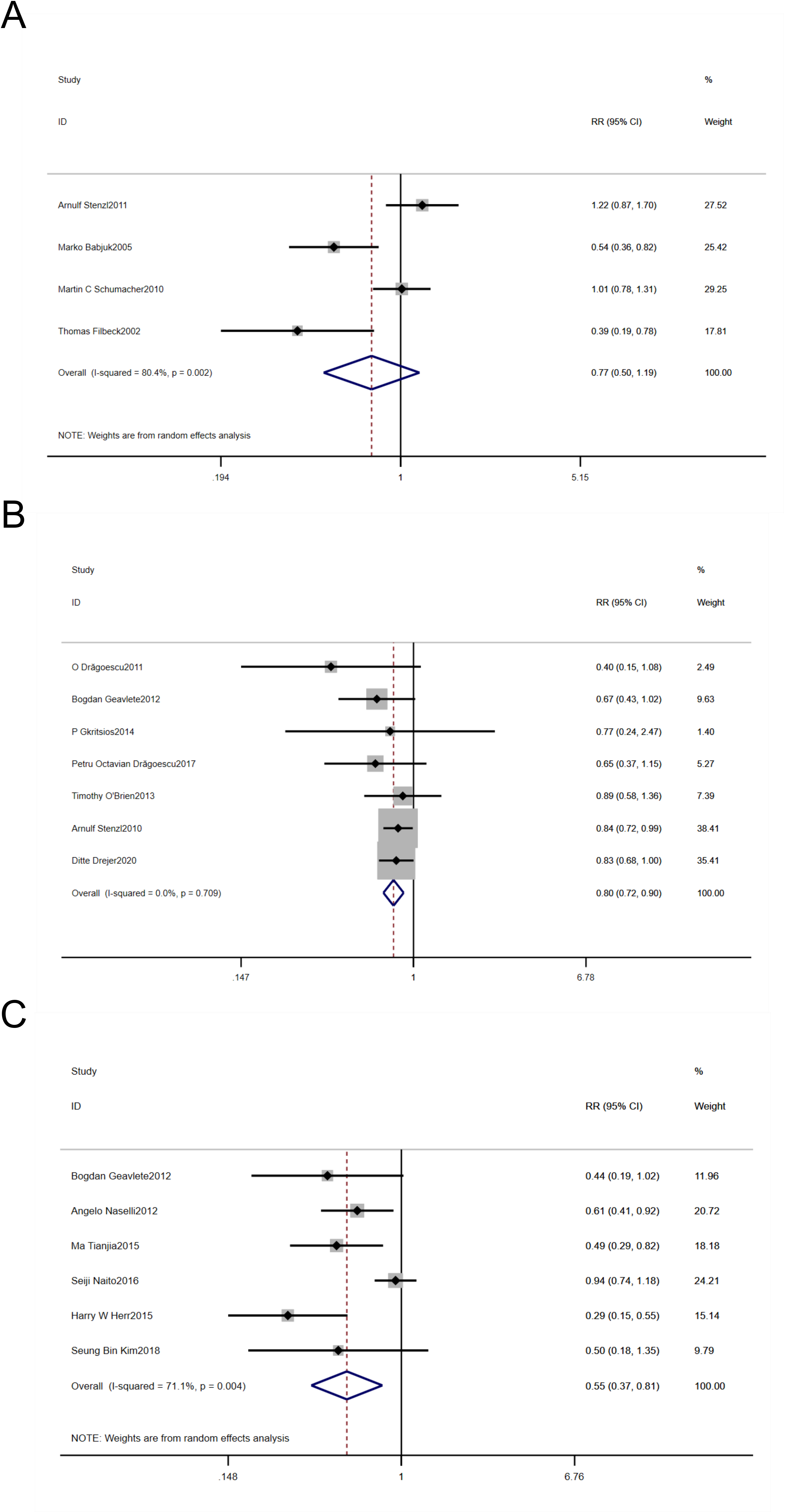
**

**[Supplementary Fig. 1](https://www.ncbi.nlm.nih.gov/pmc/articles/PMC9913451/#app1-cancers-15-00600)1.** Subgroup analysis(intravesical chemotherapy): Forest plots of long-term recurrence rate of bladder cancer in pairwise meta-analysis. (A) 5-ALA VS WLC, (B) HAL VS WLC, (C) NBI VS WLC. 5-ALA: 5-aminolevulinic acid; HAL: hexaminolaevu-linate; NBI:narrow band imaging; WIC:white light cystoscopy.**
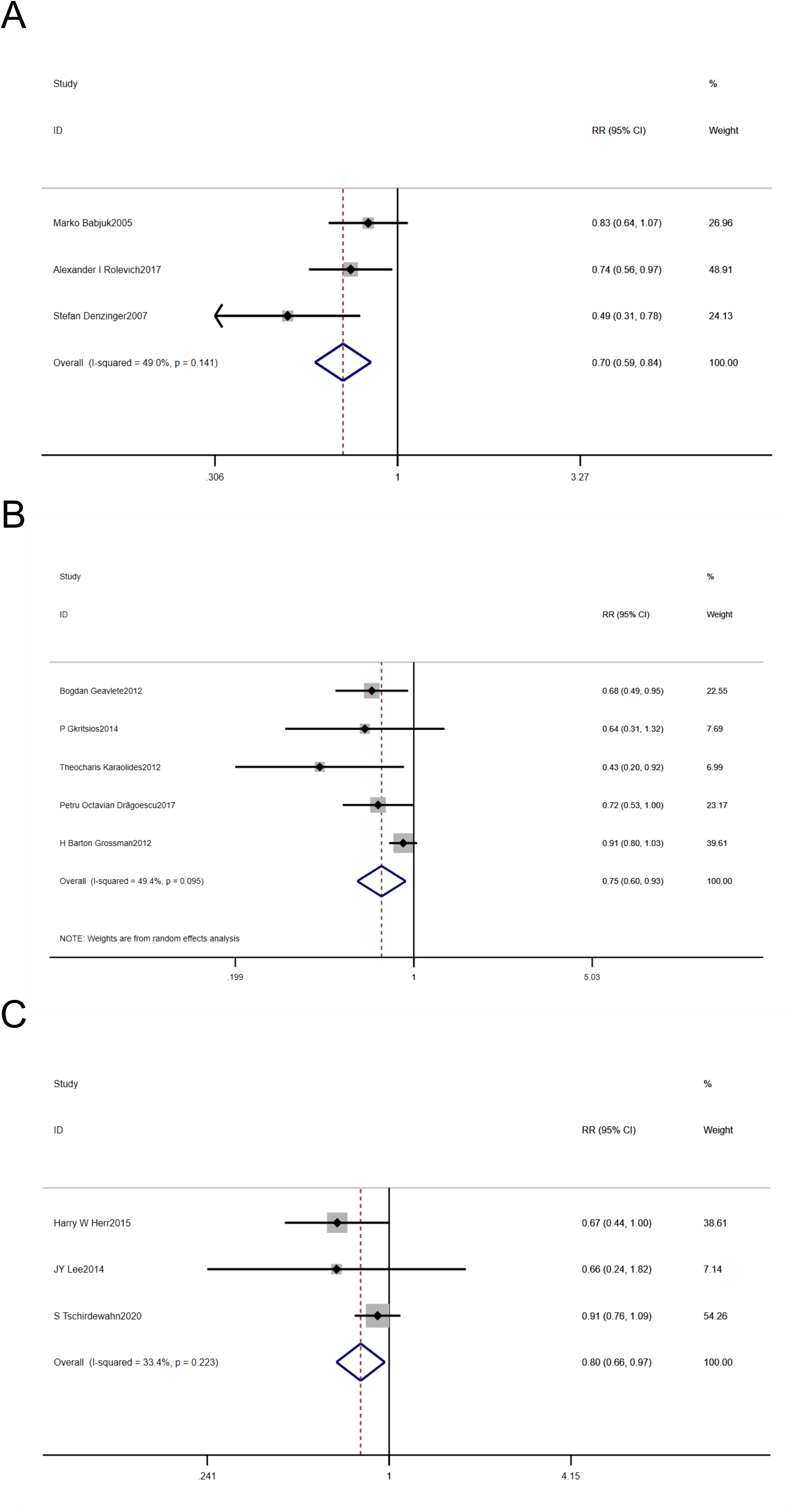
**

**[Supplementary Fig. 1](https://www.ncbi.nlm.nih.gov/pmc/articles/PMC9913451/#app1-cancers-15-00600)2.** Subgroup analysis(second TURBT): Forest plots of short-term recurrence rate of bladder cancer in pairwise meta-analysis. (A) 5-ALA VS WLC, (B) HAL VS WLC, (C) NBI VS WLC. 5-ALA: 5-aminolevulinic acid; HAL: hexaminolaevu-linate; NBI:narrow band imaging; WIC:white light cystoscopy.

**
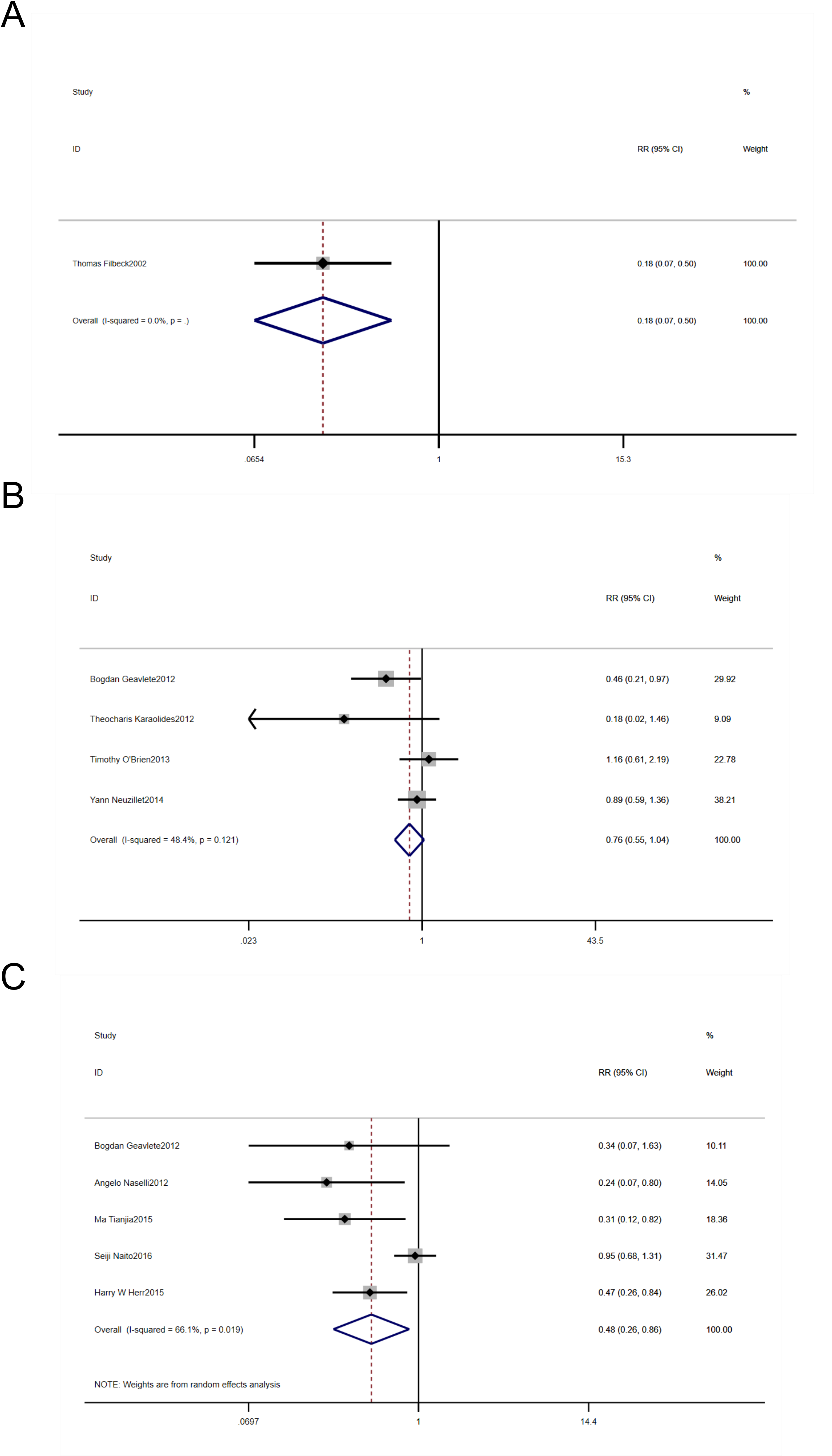
**

**[Supplementary Fig. 1](https://www.ncbi.nlm.nih.gov/pmc/articles/PMC9913451/#app1-cancers-15-00600)3.** Subgroup analysis(second TURBT): Forest plots of intermediate-term recurrence rate of bladder cancer in pairwise meta-analysis. (A) 5-ALA VS WLC, (B) HAL VS WLC, (C) NBI VS WLC. 5-ALA: 5-aminolevulinic acid; HAL: hexaminolaevu-linate; NBI:narrow band imaging; WIC:white light cystoscopy.

**
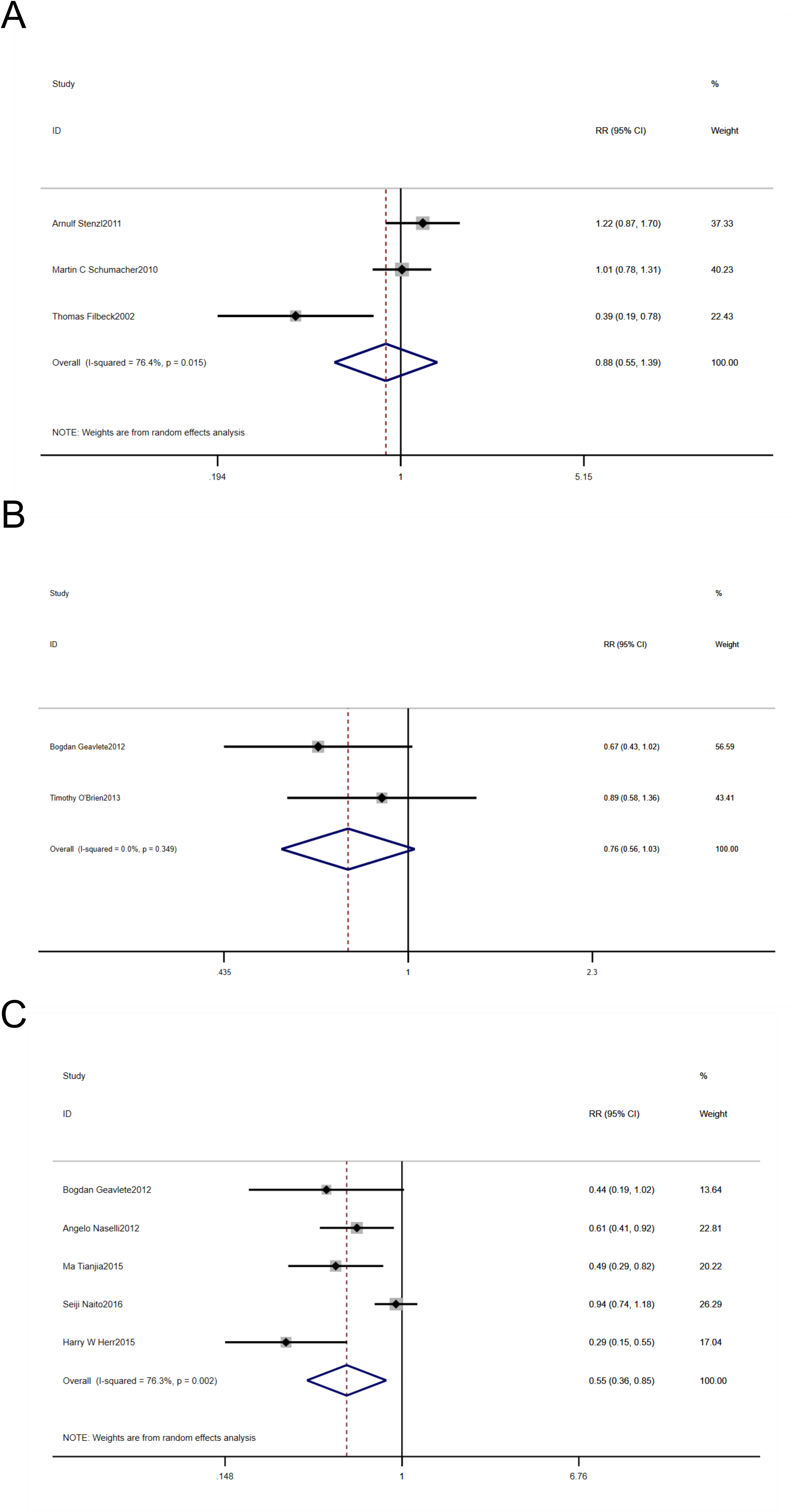
**

**[Supplementary Fig. 1](https://www.ncbi.nlm.nih.gov/pmc/articles/PMC9913451/#app1-cancers-15-00600)4.** Subgroup analysis(second TURBT): Forest plots of long-term recurrence rate of bladder cancer in pairwise meta-analysis. (A) 5-ALA VS WLC, (B) HAL VS WLC, (C) NBI VS WLC. 5-ALA: 5-aminolevulinic acid; HAL: hexaminolaevu-linate; NBI:narrow band imaging; WIC:white light cystoscopy.

**
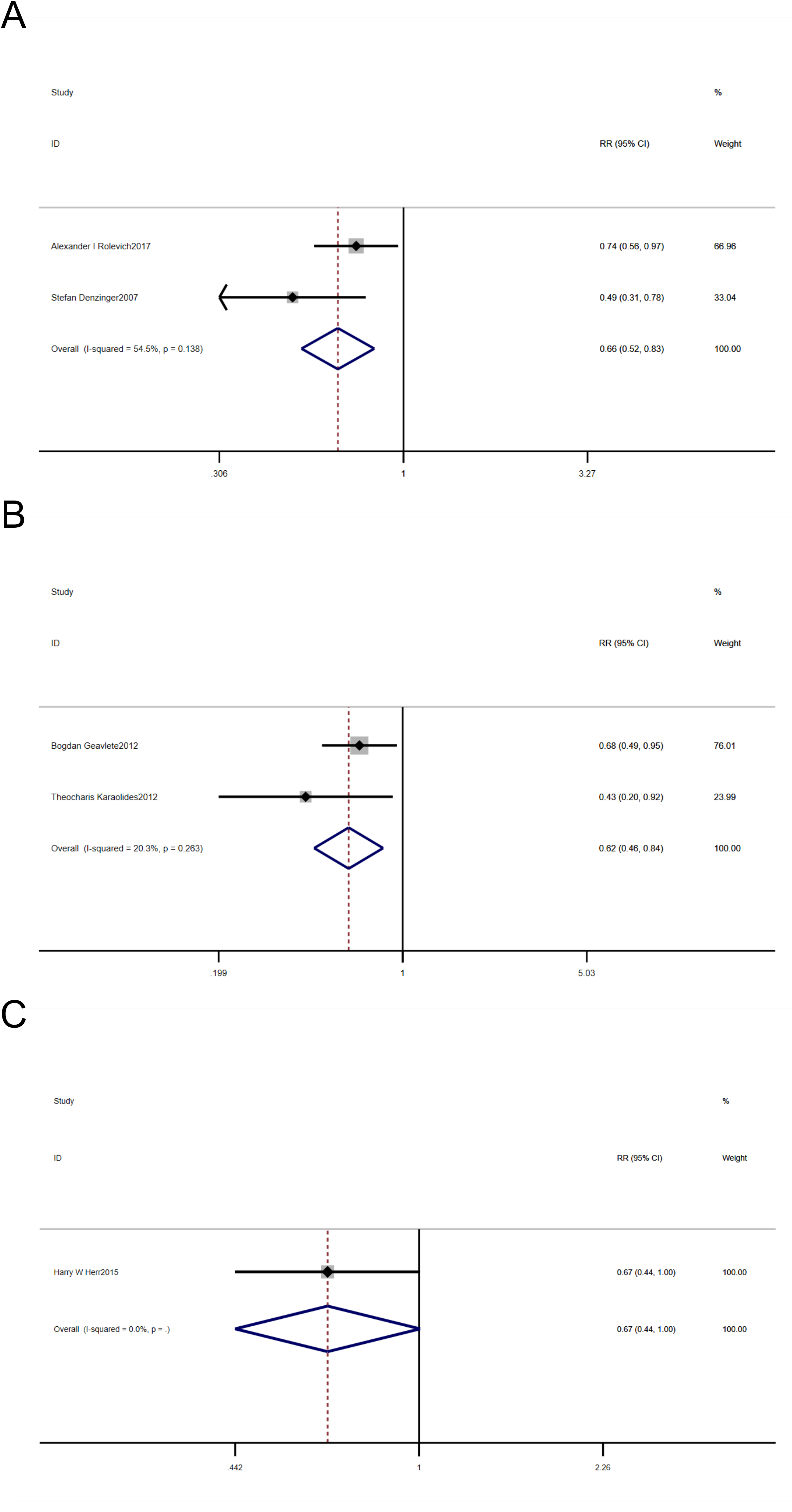
**

**[Supplementary Fig. 1](https://www.ncbi.nlm.nih.gov/pmc/articles/PMC9913451/#app1-cancers-15-00600)5.** The area under the SROC curve. (A) 5-ALA, (B) HAL, (C) NBI, (D) WLC. SROC:summary receiver operating characteristic, 5-ALA: 5-aminolevulinic acid, HAL: hexaminolaevu-linate, NBI:narrow band imaging, WIC:white light cystoscopy.

**
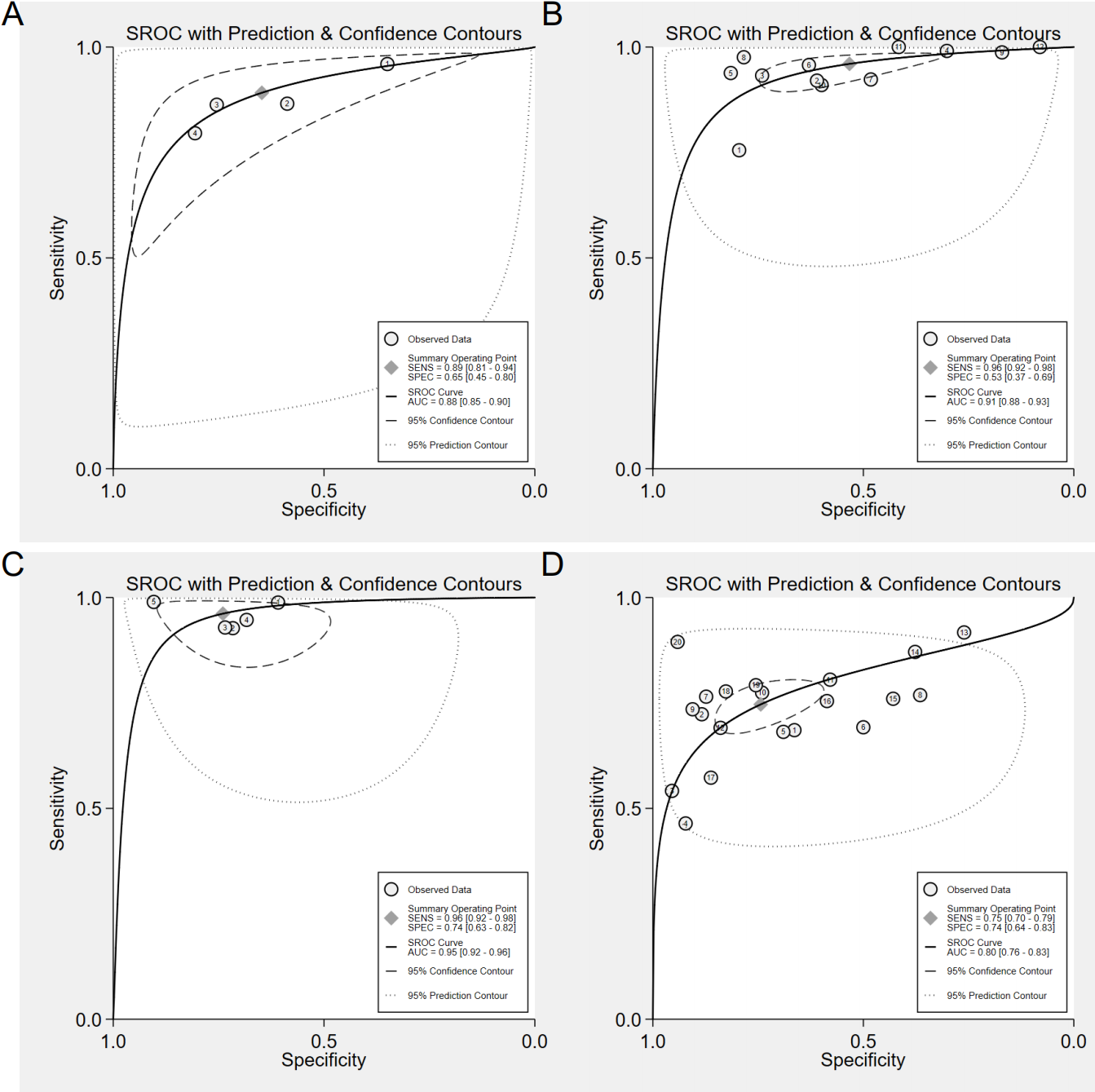
**

**[Supplementary Fig. 1](https://www.ncbi.nlm.nih.gov/pmc/articles/PMC9913451/#app1-cancers-15-00600)6.** Forest plots of detection rate of Ta stage bladder tumors in pairwise meta-analysis. (A) 5-ALA VS WLC, (B) HAL VS WLC, (C) NBI VS WLC. 5-ALA: 5-aminolevulinic acid, HAL: hexaminolaevu-linate, NBI:narrow band imaging, WIC:white light cystoscopy.

**
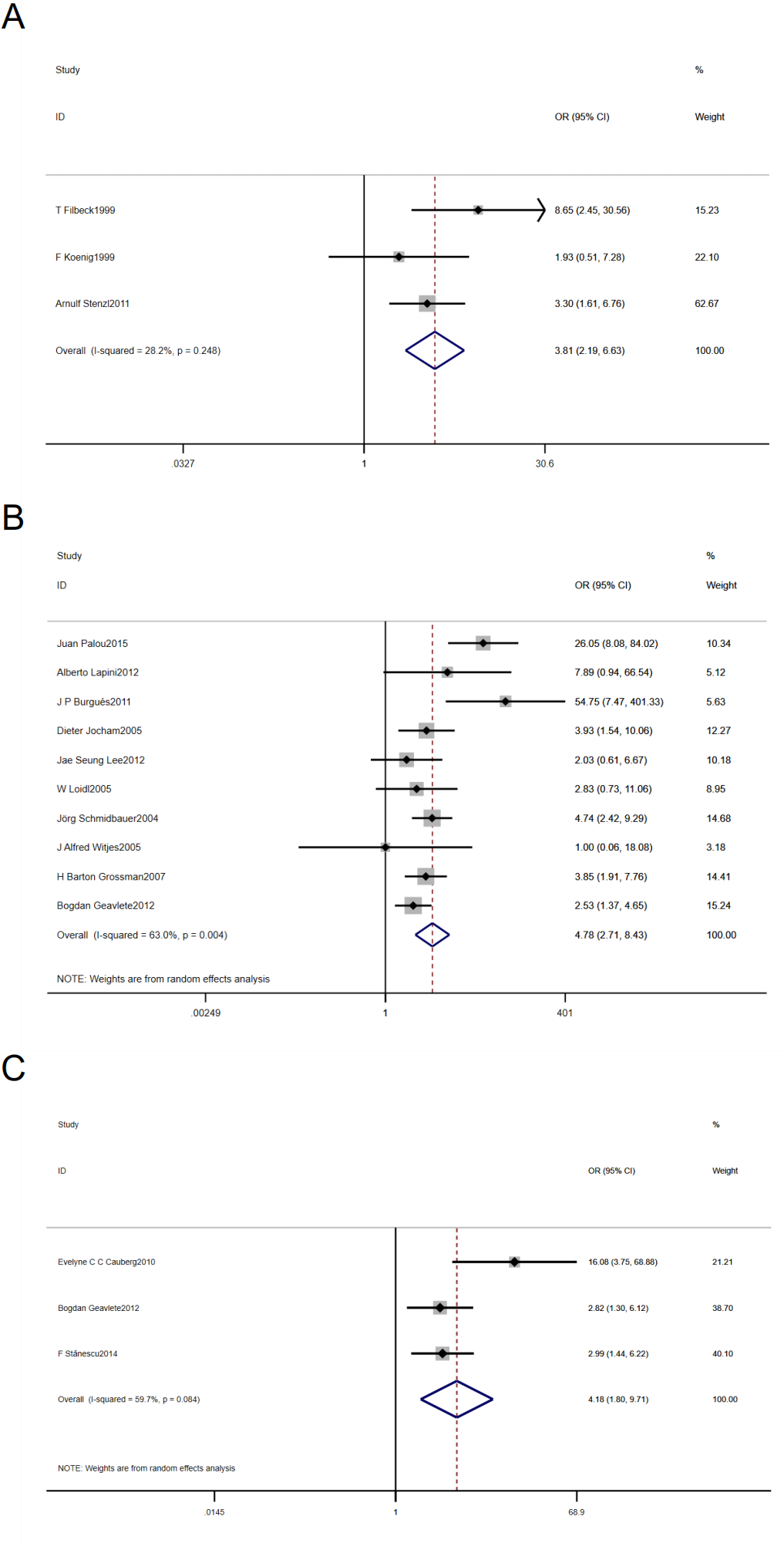
**

**[Supplementary Fig. 1](https://www.ncbi.nlm.nih.gov/pmc/articles/PMC9913451/#app1-cancers-15-00600)7.** Forest plots of detection rate of T1 stage bladder tumors in pairwise meta-analysis. (A) 5-ALA VS WLC, (B) HAL VS WLC, (C) NBI VS WLC. 5-ALA: 5-aminolevulinic acid, HAL: hexaminolaevu-linate, NBI:narrow band imaging, WIC:white light cystoscopy.

**
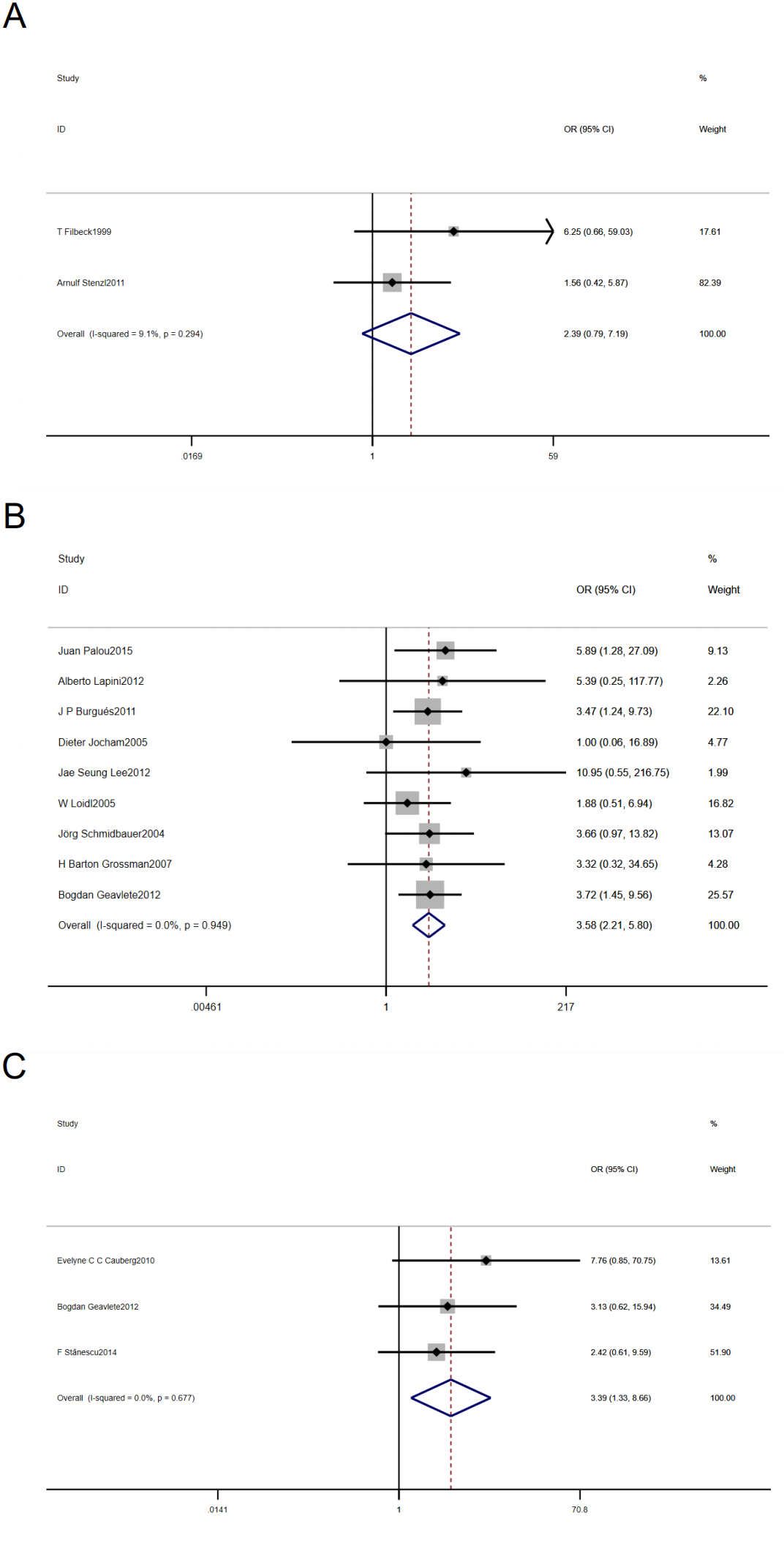
**

**[Supplementary Fig. 1](https://www.ncbi.nlm.nih.gov/pmc/articles/PMC9913451/#app1-cancers-15-00600)8.** Forest plots of detection rate of CIS in pairwise meta-analysis. (A) 5-ALA VS WLC, (B) HAL VS WLC, (C) NBI VS WLC. 5-ALA: 5-aminolevulinic acid, HAL: hexaminolaevu-linate, NBI:narrow band imaging, WIC:white light cystoscopy; CIS:carcinoma in situ.

**
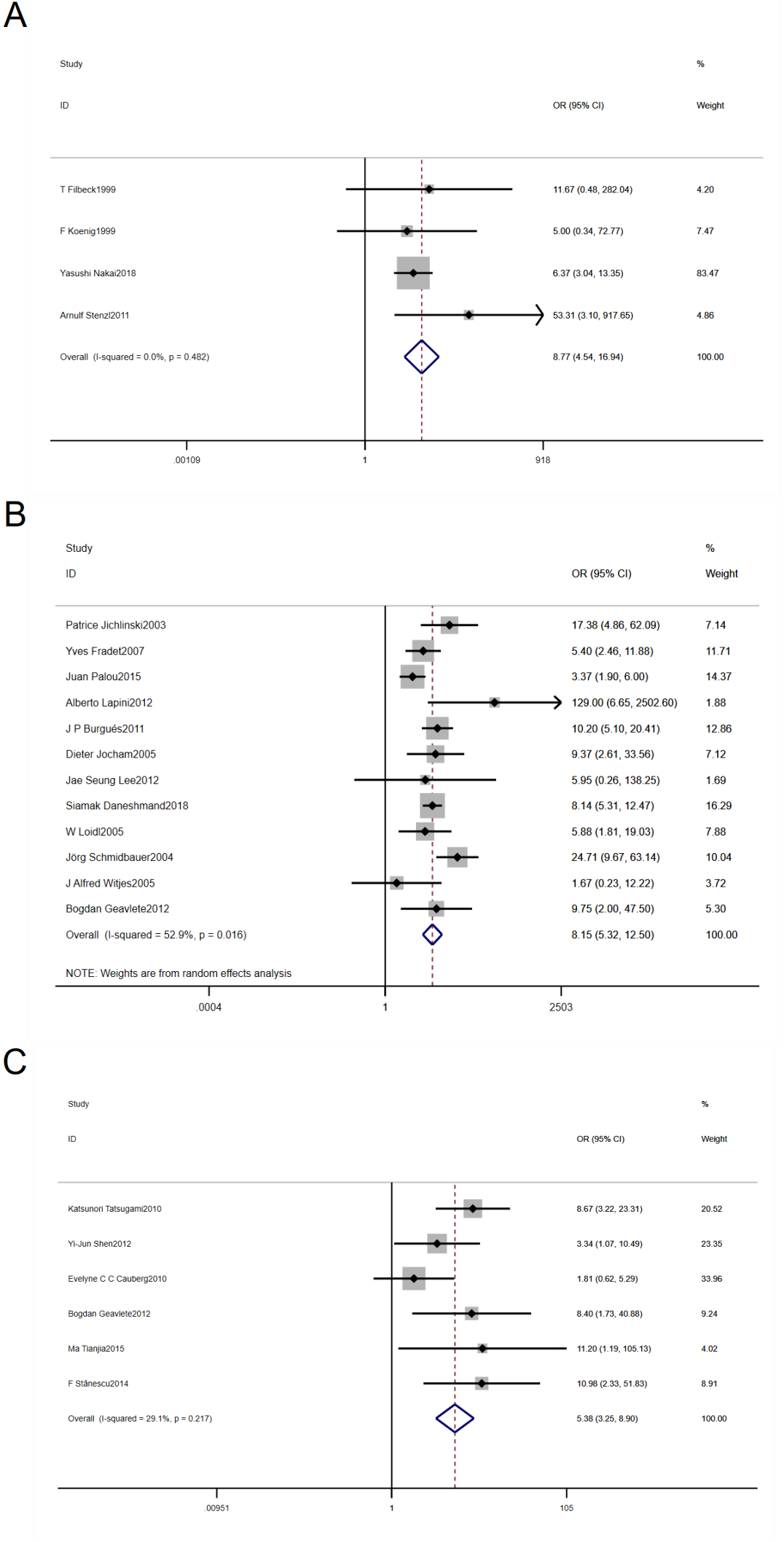
**

**[Supplementary Fig. 1](https://www.ncbi.nlm.nih.gov/pmc/articles/PMC9913451/#app1-cancers-15-00600)9.** Forest plots of detection rate of muscle-invasive bladder cancer in pairwise meta-analysis. (A) 5-ALA VS WLC, (B) HAL VS WLC, (C) NBI VS WLC. 5-ALA: 5-aminolevulinic acid, HAL: hexaminolaevu-linate, NBI:narrow band imaging, WIC:white light cystoscopy.

**
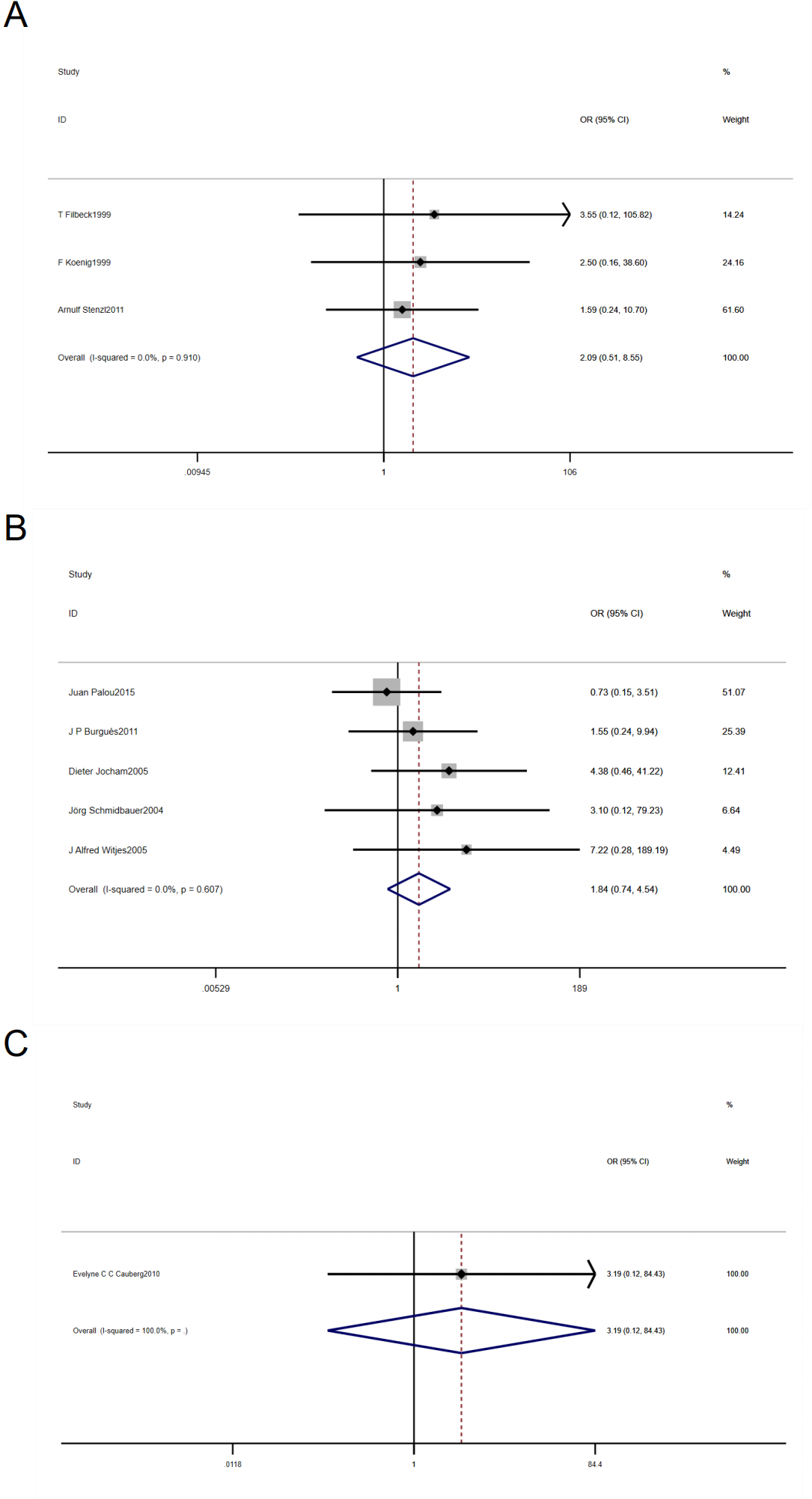
**

**[Supplementary Fig. 2](https://www.ncbi.nlm.nih.gov/pmc/articles/PMC9913451/#app1-cancers-15-00600)0.** Forest plots of short-term progression rate of bladder cancer in pairwise meta-analysis. (A) 5-ALA VS WLC, (B) HAL VS WLC, (C) NBI VS WLC. 5-ALA: 5-aminolevulinic acid, HAL: hexaminolaevu-linate, NBI:narrow band imaging, WIC:white light cystoscopy.

**
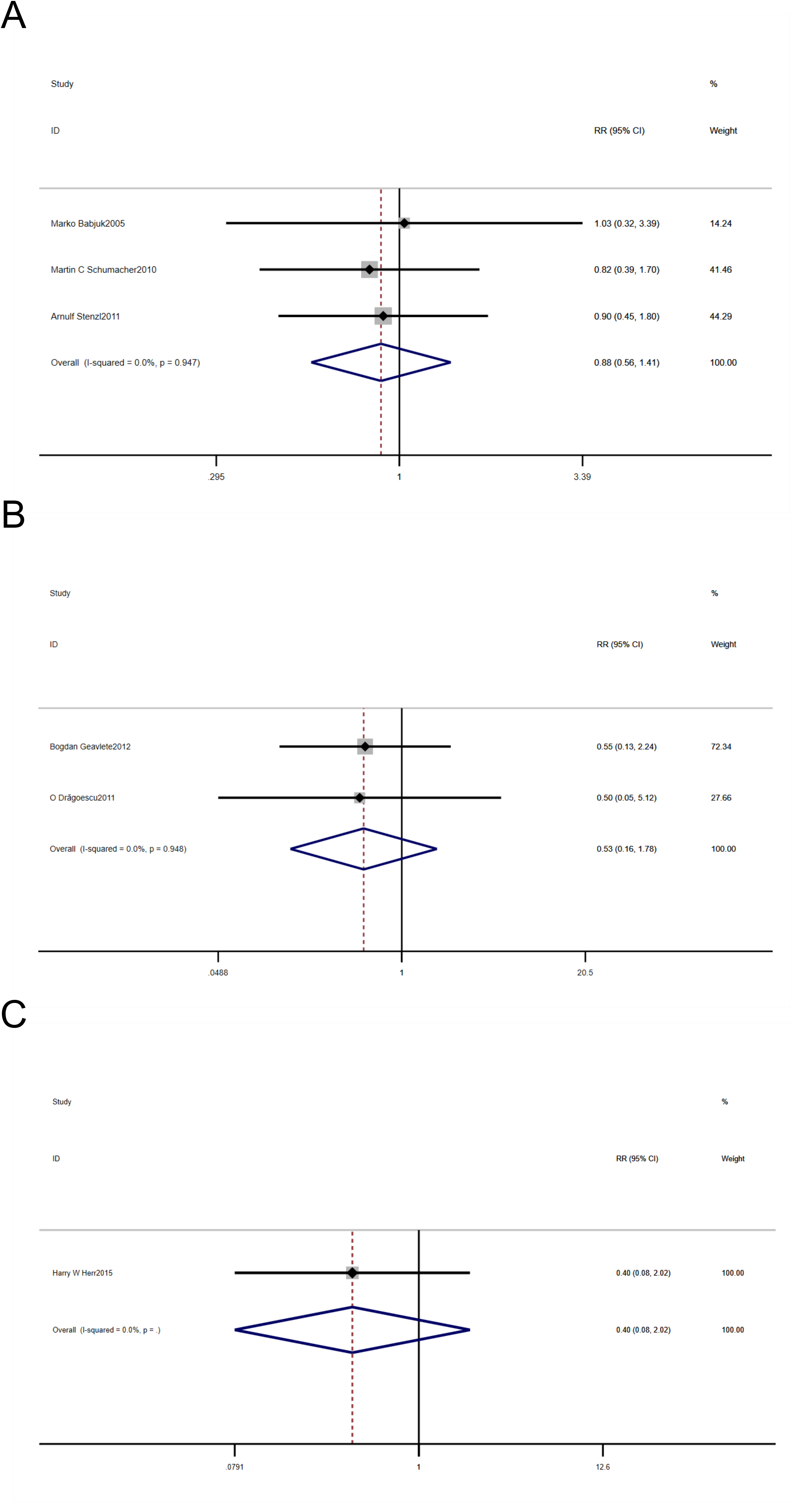
**

**[Supplementary Fig. 2](https://www.ncbi.nlm.nih.gov/pmc/articles/PMC9913451/#app1-cancers-15-00600)1.** Forest plots of long-term progression rate of bladder cancer in pairwise meta-analysis. (A) 5-ALA VS WLC, (B) HAL VS WLC, (C) NBI VS WLC. 5-ALA: 5-aminolevulinic acid, HAL: hexaminolaevu-linate, NBI:narrow band imaging, WIC:white light cystoscopy.


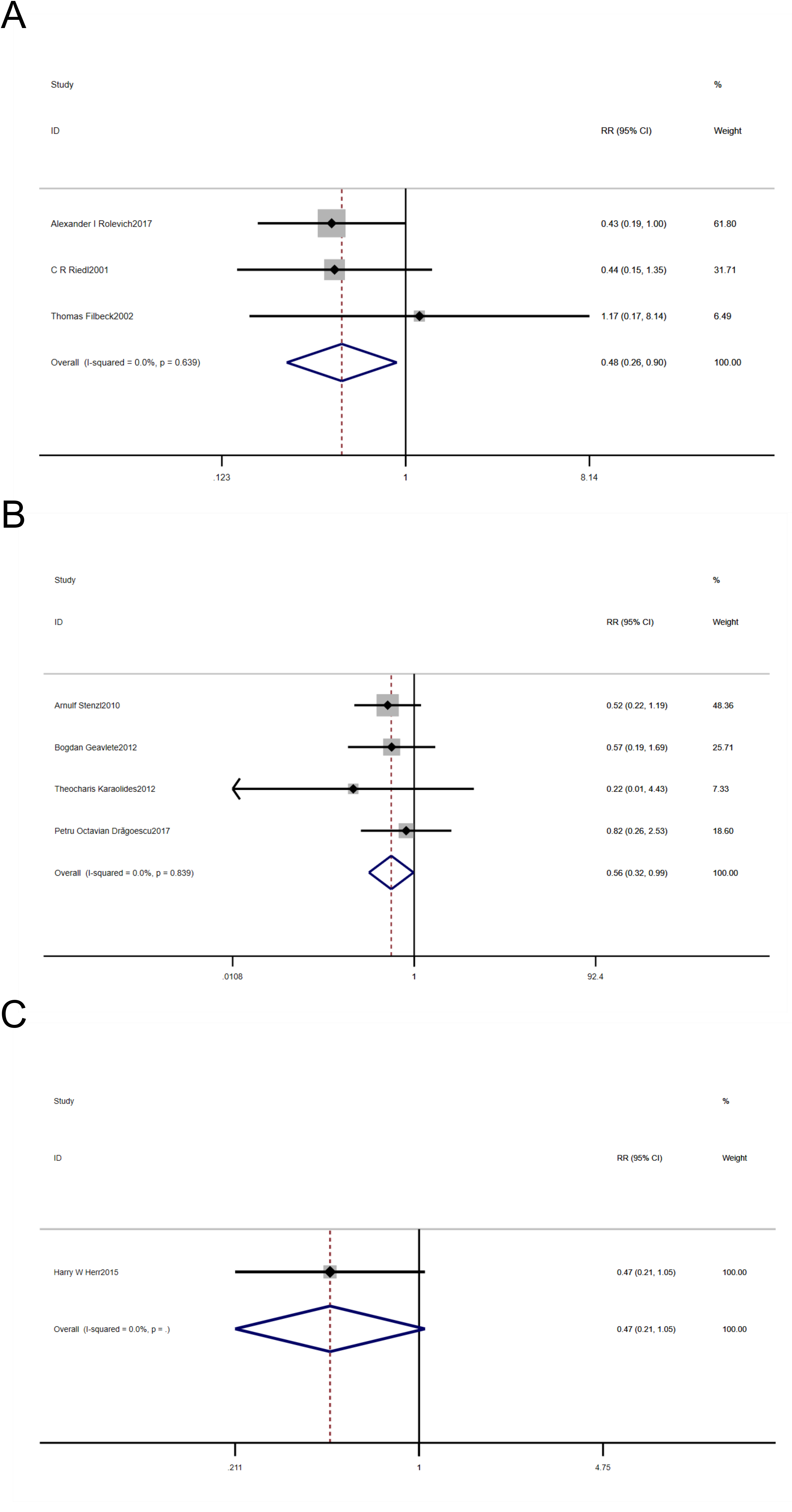


**[Supplementary Fig. 2](https://www.ncbi.nlm.nih.gov/pmc/articles/PMC9913451/#app1-cancers-15-00600)2.** Funnel plots of recurrence rate of bladder cancer in network meta-analysis. (A) Short-term recurrence rate, egger's test: 0.001, (B) Intermediate-term recurrence rate, egger's test: 0.002, (C) Long-term recurrence rate, egger's test: 0.001. 5-ALA: 5-aminolevulinic acid, HAL: hexaminolaevu-linate, NBI:narrow band imaging, WIC:white light cystoscopy.

**
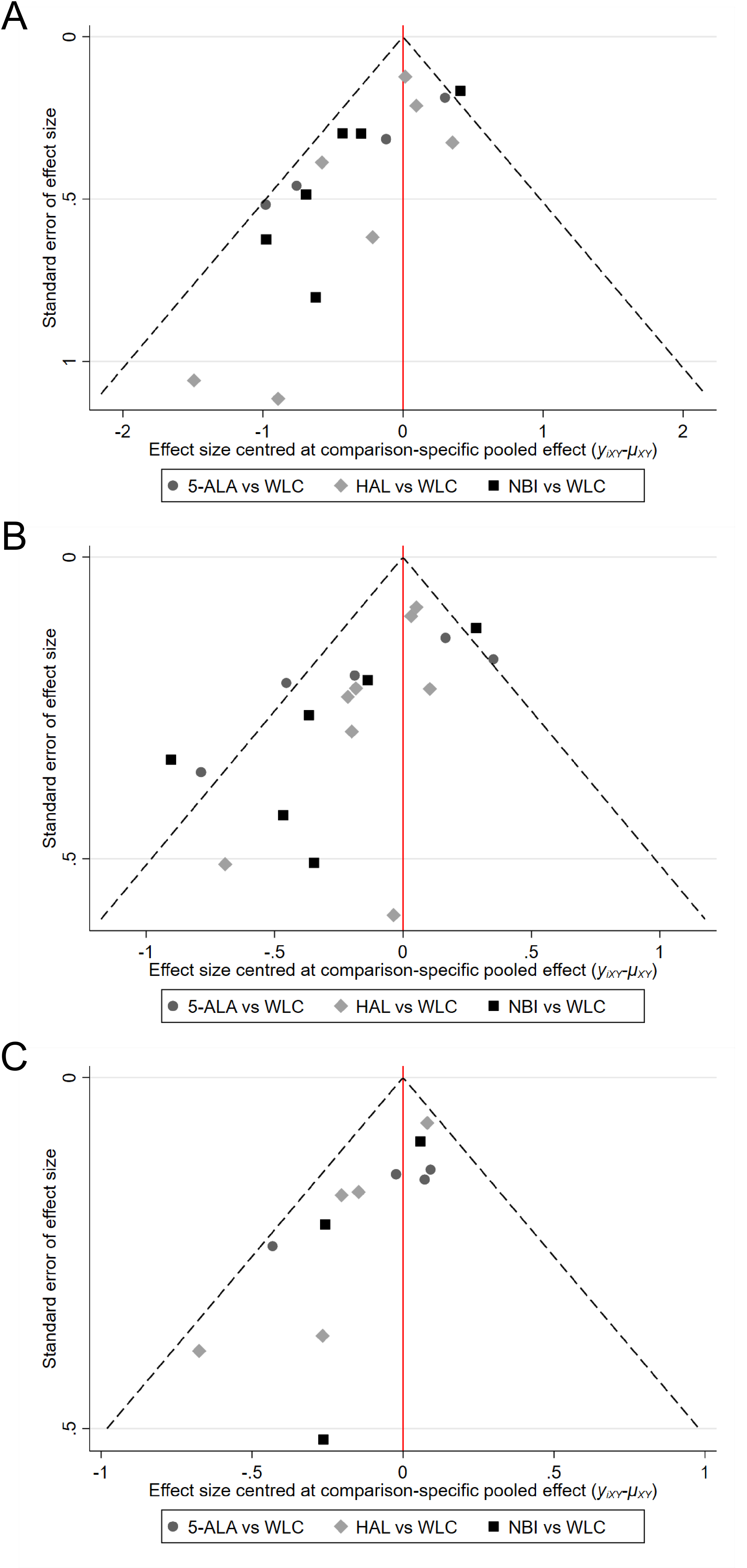
**

**[Supplementary Fig. 2](https://www.ncbi.nlm.nih.gov/pmc/articles/PMC9913451/#app1-cancers-15-00600)3.** Funnel plots of detection rate of bladder cancer in network meta-analysis. (A) Ta stage tumors, egger's test: 0.139, (B) T1 stage tumors, egger's test: 0.413, (C) CIS, egger's test: 0.386. 5-ALA: 5-aminolevulinic acid, HAL: hexaminolaevu-linate, NBI:narrow band imaging, WIC:white light cystoscopy; CIS:carcinoma in situ.

**
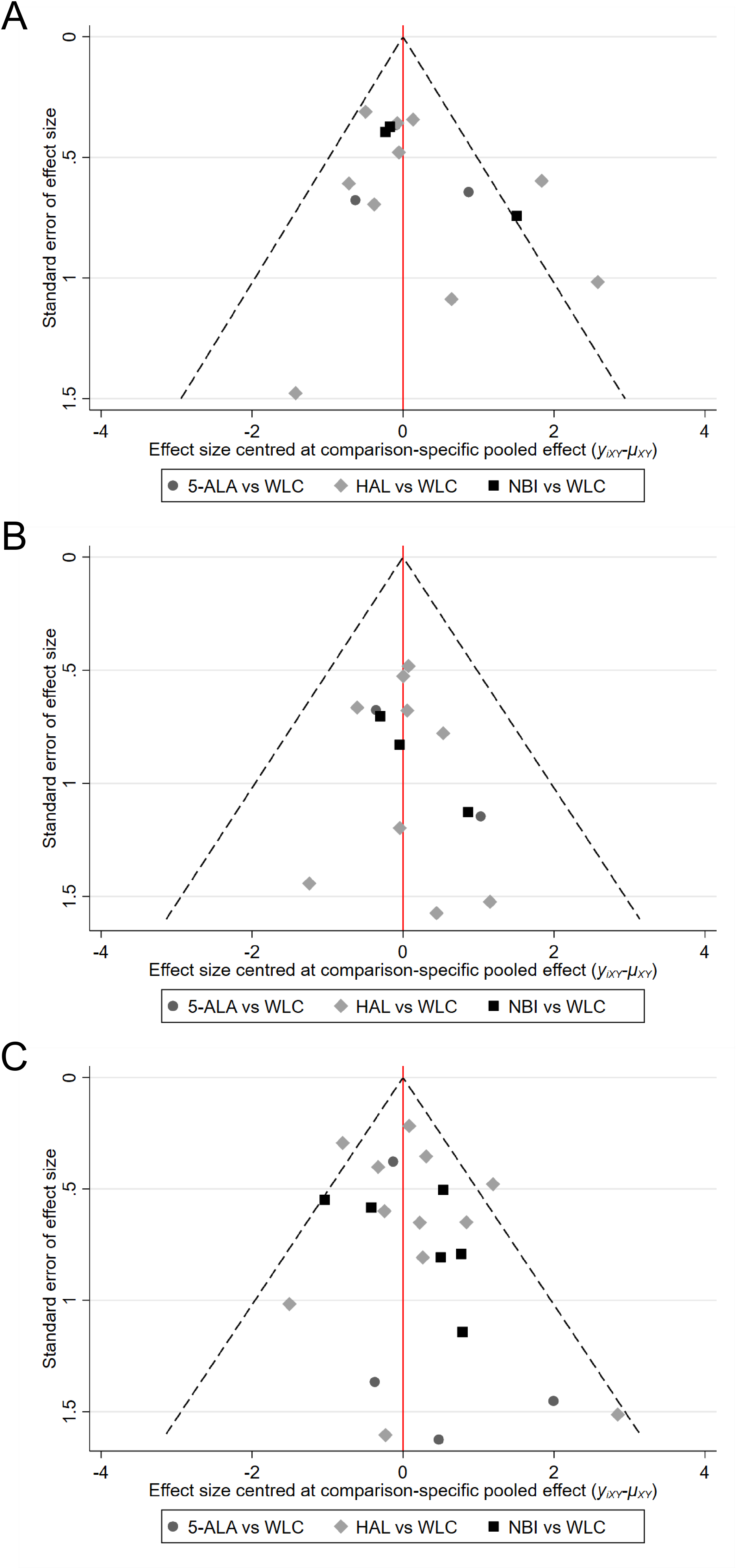
**

**[Supplementary Fig. 2](https://www.ncbi.nlm.nih.gov/pmc/articles/PMC9913451/#app1-cancers-15-00600)4.** Funnel plots of detection rate of bladder cancer in pairwise meta-analysis. (A) HAL VS WLC in Ta stage tumors, egger's test: 0.363, (B) HAL VS WLC in CIS, egger's test: 0.451. HAL: hexaminolaevu-linate, WIC:white light cystoscopy; CIS:carcinoma in situ.

**
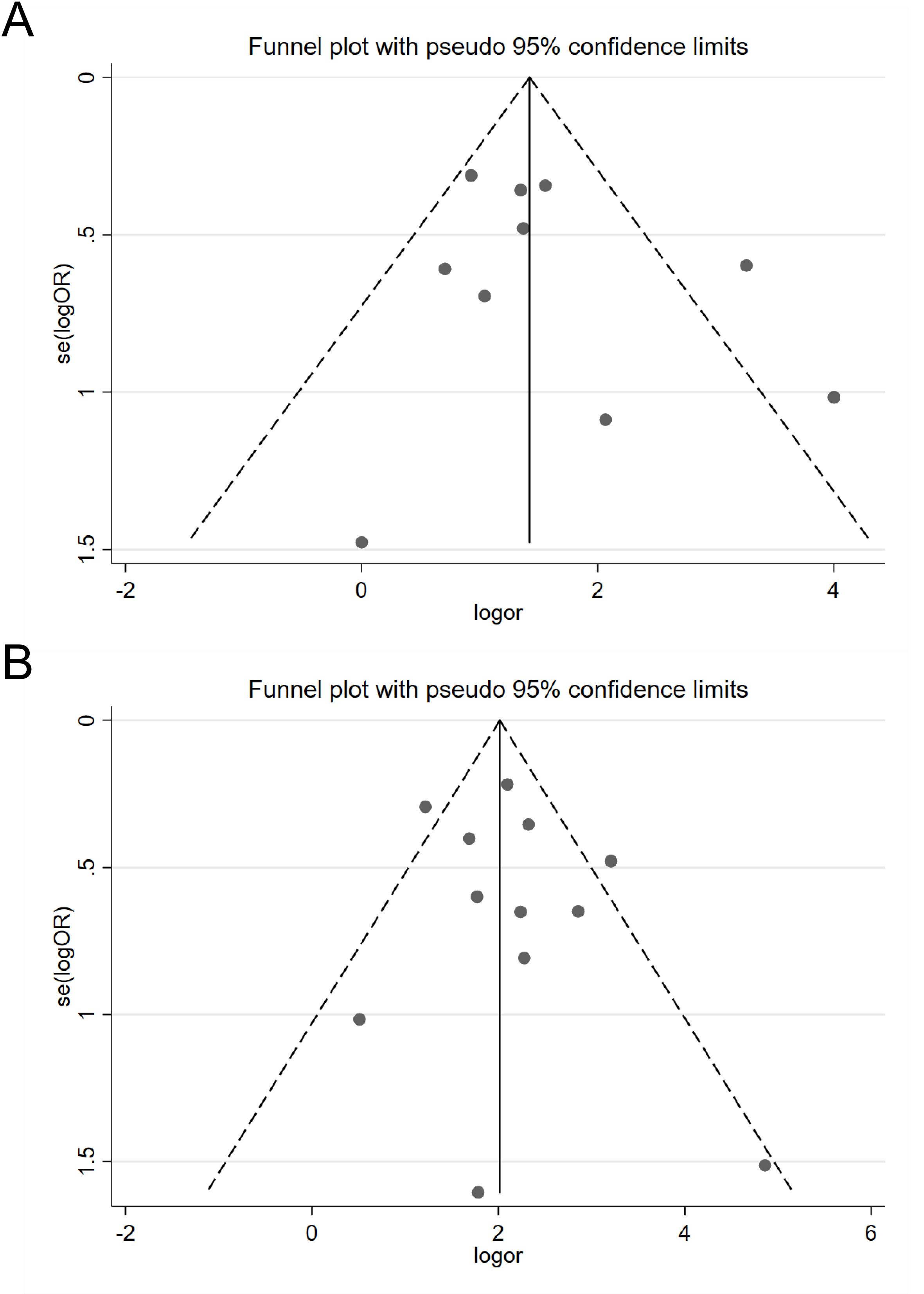
**
